# The Role of Circulating MicroRNAs as Biomarkers and Therapeutic Targets in Venous Thromboembolism: A Systematic Review

**DOI:** 10.3390/jcm15166306

**Published:** 2026-08-14

**Authors:** Bulent Kantarcioglu, Hande Nur Erölmez, Mira Nigudkar, Martin Lundy, Fakiha Siddiqui, Prakasha Kempaiah, Chongyu Zhang, Grigoris Gerotziafas, Walter Keith Jones, Jawed Fareed

**Affiliations:** 1 Department of Internal Medicine, Division of Hematology, University of Health Sciences, Sancaktepe Sehit Prof. Dr. Ilhan Varank, Training and Research Hospital, 34785 Istanbul, Türkiye; 2 Department of Pathology and Laboratory Medicine, Health Sciences Division, Cardiovascular Research Institute, Loyola University Chicago, Maywood, IL 60153, USA; fsiddiqui3@luc.edu (F.S.); jfareed@luc.edu (J.F.); 3 Department of Molecular Pharmacology and Neuroscience, Health Sciences Division, Loyola University Chicago, Maywood, IL 60153, USA; pkempaiah@luc.edu (P.K.); czhang13@luc.edu (C.Z.); wjones7@luc.edu (W.K.J.); 4 Department of Family Medicine, University of Health Sciences, Umraniye Training and Research Hospital, 34764 Istanbul, Türkiye; herolmez@gmail.com; 5 Department of Medicine, Loyola University Stritch School of Medicine, Chicago, IL 60153, USA; mnigudkar@luc.edu (M.N.); mlundy1@luc.edu (M.L.); 6 Sorbonne Université, Assistance Publique-Hôpitaux de Paris, Thrombosis Center, Service D’Hématologie Biologique Hôpital Tenon, 75005 Paris, France; grigorios.gerotziafas@inserm.fr

**Keywords:** miRNA, deep vein thrombosis, pulmonary embolism, venous thromboembolism, biomarker

## Abstract

**Introduction:** Venous thromboembolism (VTE), encompassing deep vein thrombosis (DVT) and pulmonary embolism (PE), is a major cardiovascular disorder and a leading cause of vascular mortality. MicroRNAs (miRNAs) are small endogenous noncoding RNAs that regulate gene expression post-transcriptionally by binding to the target messenger RNAs (mRNAs), inducing mRNA degradation or translational repression. miRNA dysregulation likely contributes to VTE pathogenesis. This review comprehensively summarizes current evidence on miRNAs in VTE, offering a structured overview of the existing literature. **Materials and Methods:** Eligibility criteria were defined using the Population, Intervention/Exposure, Comparator, Outcomes, and Study design (PICOS) framework. A comprehensive literature search in PubMed and EMBASE was conducted in accordance with Preferred Reporting Items for Systematic Reviews and Meta-Analyses (PRISMA) guidelines. Given methodological heterogeneity, a narrative synthesis was undertaken. **Results:** Among the 156 studies included in this review, 99 investigated miRNAs in DVT, 25 examined the role of miRNAs in PE, and 32 evaluated miRNA involvement in VTE. These miRNAs were categorized as prothrombotic, antithrombotic, or unclassified based on their reported functional roles. **Conclusions:** This systematic review highlights the impact of miRNAs in the pathophysiology of VTE and provides a resource for future mechanistic and translational research. The findings emphasize the need for standardized, large-scale studies focusing on human-specific models to validate miRNAs as diagnostic, prognostic biomarkers, and as therapeutic targets in VTE.

## 1. Introduction

Venous thromboembolism (VTE), encompassing deep vein thrombosis (DVT) and pulmonary embolism (PE), is a major cardiovascular disorder and a leading cause of vascular mortality [1]. Its incidence is 1–2 per 1000 person-years, with a lifetime risk of approximately 8%, affecting over 1.2 million individuals annually in the United States and approximately 10 million worldwide [2,3,4,5,6]. Recent nationwide data from China demonstrated an increasing incidence and serious mortality burden of VTE, challenging the traditional perception that VTE is markedly less common in Asian populations than in Western countries [7]. PE is the most severe presentation, accounting for the majority of VTE-related deaths and an approximately 20% one-year mortality rate [8,9]. VTE also confers substantial long-term morbidity: approximately 30% of patients experience recurrence within 10 years, and complications including post-thrombotic syndrome (PTS), post-pulmonary embolism syndrome, anticoagulant-related bleeding, and chronic thromboembolic pulmonary hypertension (CTEPH) impair quality of life and increase healthcare utilization [10,11,12,13,14,15]. The annual economic burden reaches €1.5–3.3 billion in Europe and US$7–10 billion in the United States [16,17].

Biomarkers have an established but complementary role in the diagnosis and risk stratification of VTE. D-dimer is the principal circulating biomarker used in the diagnostic evaluation of suspected VTE and, because of its high sensitivity, is particularly useful for excluding VTE in patients with a low or intermediate clinical probability. However, its specificity is limited because D-dimer concentrations may also be elevated with increasing age and in numerous conditions, including cancer, infection, inflammation, pregnancy, trauma, and recent surgery. In patients with acute PE, cardiac troponins and natriuretic peptides, particularly B-type natriuretic peptide and N-terminal pro-B-type natriuretic peptide, are well-established additional biomarkers for assessing myocardial injury and right ventricular strain and are primarily used for prognostic risk stratification rather than diagnosis [18,19,20]. Other circulating markers reflecting inflammation, endothelial activation, myocardial stress, coagulation, and fibrinolysis have also been investigated, including C-reactive protein, heart-type fatty acid-binding protein, soluble P-selectin, von Willebrand factor, and several coagulation and fibrinolytic mediators. However, none has replaced D-dimer in routine VTE diagnostic algorithms or provided sufficient specificity and reproducibility for broad clinical application [18,19,20].

These limitations have stimulated the search for biomarkers that may more specifically reflect the biological processes underlying VTE and potentially provide information beyond the presence of fibrin formation and degradation. Circulating microRNAs (miRNAs) have emerged as promising epigenetic biomarker candidates because they are detectable in blood, exhibit relative stability in the circulation, and can be quantified using sensitive molecular techniques. Their stability is enhanced by their association with extracellular vesicles, lipoproteins, and RNA-binding proteins, which protect them from degradation. In contrast to conventional protein biomarkers that often represent downstream consequences of thrombosis, altered miRNA expression may reflect upstream and interconnected biological processes involved in thrombogenesis, including endothelial dysfunction, platelet activation, inflammation, coagulation, and fibrinolysis. Furthermore, individual miRNAs or miRNA signatures may provide disease-related molecular information and potentially improve diagnostic, prognostic, or phenotypic characterization when used in combination with established clinical and laboratory approaches. These potential advantages have prompted increasing investigation of circulating miRNAs as biomarkers of VTE, although their clinical utility remains to be established through standardized assays and prospective external validation.

miRNAs are small endogenous noncoding RNAs (19–24 nucleotides) that regulate gene expression post-transcriptionally by binding to the 3′ untranslated region of target messenger RNAs (mRNAs), thereby inducing mRNA degradation or translational repression [21]. A single miRNA may regulate multiple genes, collectively influencing a large proportion of the human transcriptome (approximately 90%) and controlling processes including cell-cycle regulation, apoptosis, proliferation, and differentiation [22]. miRNAs are key regulators in numerous diseases [23,24] and can be detected in body fluids by Northern blotting, quantitative real-time polymerase chain reaction (qRT-PCR), microarray analysis, and next-generation sequencing [25]. Aberrant miRNA expression has been linked to cardiovascular, neoplastic, and autoimmune conditions [26,27]. In hemostasis, miRNAs modulate endothelial cells, platelets, and leukocytes and regulate procoagulants, including fibrinogen, factor XI, and tissue factor (TF); anticoagulants, including tissue factor pathway inhibitor, antithrombin, and protein S; and fibrinolytic mediators, including plasminogen activator inhibitor-1 (PAI-1) [28,29,30]. Given these broad regulatory roles, miRNA dysregulation may contribute to VTE pathogenesis while also providing measurable signatures of the underlying thrombotic processes.

Despite the rapid growth of this field, systematic syntheses of the available evidence remain limited. This systematic review aimed to comprehensively evaluate the current evidence regarding miRNAs in VTE, with particular emphasis on their mechanistic roles, circulating biomarker potential, and possible therapeutic relevance, while distinguishing promising translational candidates from findings that remain predominantly preclinical.

## 2. Materials and Methods

Eligibility criteria were defined a priori according to Population, Intervention/Exposure, Comparator, Outcomes, and Study design. Inclusion criteria were: (i) Population—laboratory systems (cell lines, primary cells, tissues, and animal models) or human participants comprising patients with VTE, healthy controls, or individuals at increased risk of VTE; (ii) Intervention/exposure—evaluation of miRNA function, expression, or diagnostic/prognostic value; (iii) Comparator—baseline versus miRNA mimic/inhibitor conditions (laboratory studies) or VTE versus non-VTE/healthy participants (clinical studies); (iv) Outcomes—cellular or molecular miRNA effects or diagnostic, prognostic, or therapeutic endpoints; (v) Study designs—in vitro, in vivo, and ex vivo experiments and cohort, case–control, or cross-sectional studies; and (vi) English-language, peer-reviewed original research. Exclusion criteria were: (i) conference abstracts, case reports, editorials, letters, narrative and systematic reviews, and meta-analyses; (ii) retracted publications; and (iii) studies focused primarily on VTE complications (PTS, CTEPH, post–pulmonary embolism syndrome) or on VTE in uncommon anatomical locations. All studies that met the predefined inclusion criteria and did not meet any exclusion criteria were included in the systematic review.

**Figure 1:**
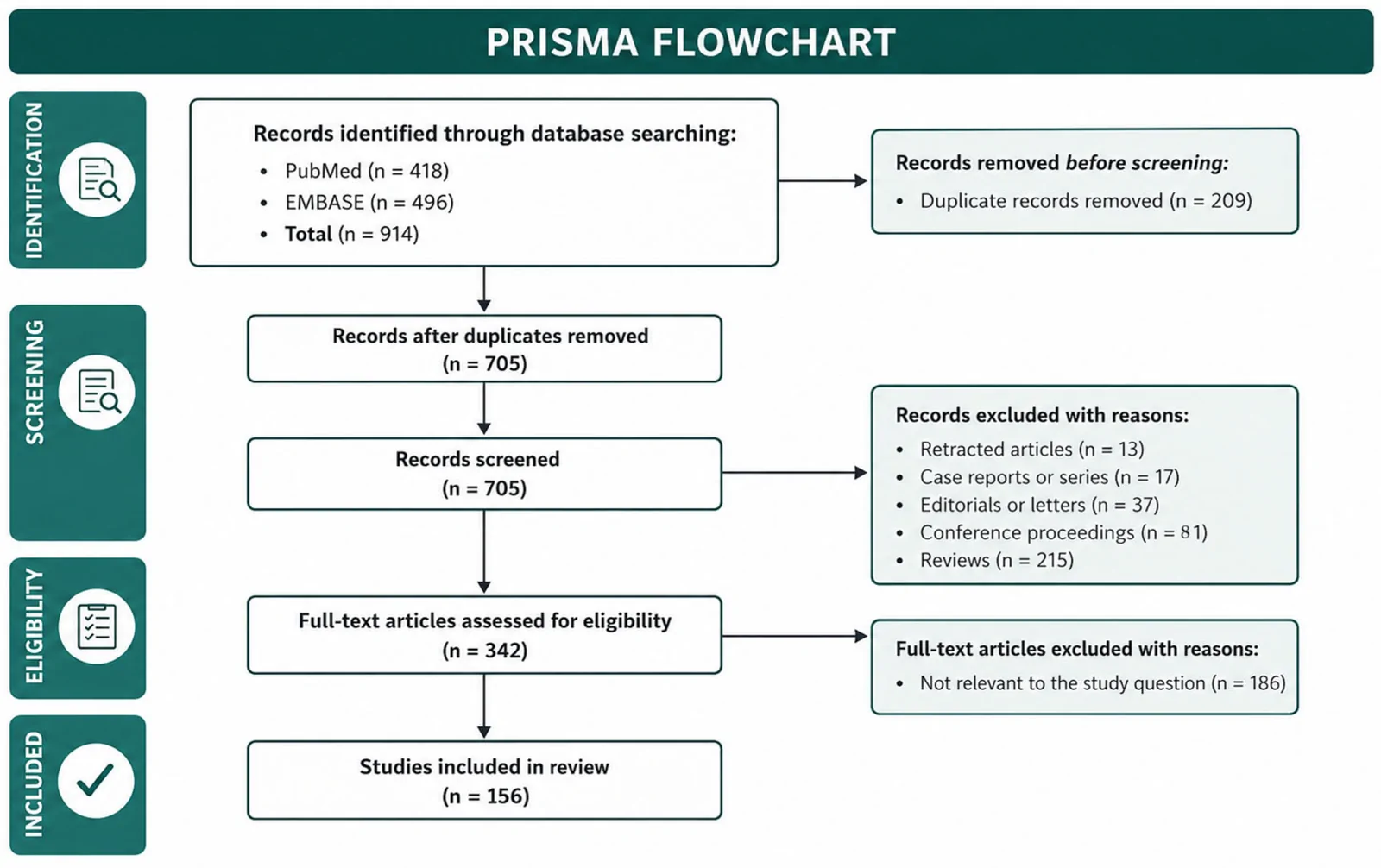
PRISMA Flowchart.

A comprehensive literature search was conducted in accordance with the Preferred Reporting Items for Systematic Reviews and Meta-Analyses (PRISMA) guidelines [31] (Figure 1). PubMed and EMBASE were queried up to 15 December 2025, using the following keywords: “miR” OR “miRNA” OR “microRNA” combined with “venous thromboembolism,” “deep vein thrombosis,” “pulmonary embolism,” “lung thrombosis,” and “lung embolism.” Reference lists of prior reviews were also screened manually. This process did not identify any additional eligible publications. PubMed and EMBASE were selected as the primary information sources because, in combination, they provide comprehensive indexing of the biomedical and pharmacological literature pertinent to miRNAs in venous thromboembolism. Two independent reviewers screened titles and abstracts, with disagreements resolved by a third reviewer. Extracted data included authors, publication year, investigated miRNAs, study design, functional role, expression patterns, mechanisms of action, identified targets, and diagnostic potential. Given the methodological heterogeneity of the studies included, a narrative synthesis was undertaken, and no meta-analysis, pooled statistical analysis, or other inferential statistical methods were performed. The review was not prospectively registered, and no formal review protocol was published. The completed PRISMA 2020 checklist is provided as Supplementary Table S1.

The risk of bias of the included studies was assessed according to study design using three established tools. The assessments were independently performed by two reviewers, with disagreements resolved through discussion and, when necessary, adjudication by a third reviewer. In vitro studies were evaluated using the Quality Assessment Tool for In Vitro Studies (QUIN), which assesses potential sources of bias related to study objectives, sample-size justification, sample preparation, allocation and randomization, blinding, outcome assessment, statistical analysis, and reporting. Animal studies were assessed using the Systematic Review Centre for Laboratory Animal Experimentation (SYRCLE) risk-of-bias tool, which evaluates selection, performance, detection, attrition, reporting, and other potential sources of bias. Clinical observational studies were assessed using the Newcastle–Ottawa Scale (NOS), with the appropriate case–control or cohort version applied according to the design of each study. Because the three tools use different domains and scoring approaches, the risk-of-bias findings for in vitro, animal, and clinical studies are separately presented in Supplementary Table S2, Supplementary Table S3 and Supplementary Table S4, respectively. An overall summary of the included studies, including their key characteristics and study designs, is provided in Supplementary Table S5.

For data synthesis, miRNAs were classified as prothrombotic or antithrombotic according to the predominant net effect on thrombus formation, progression, resolution, or overall thrombus burden reported in each source study, rather than solely based on a single direct molecular mechanism. When a miRNA demonstrated pleiotropic, context-dependent, or opposing effects across experimental models, study populations, or venous thromboembolism entities, its classification was based on the predominant direction of the reported evidence. miRNAs for which the available findings did not support a consistent net prothrombotic or antithrombotic effect were categorized as unclassified. Gene symbols are italicized throughout the manuscript; the corresponding encoded proteins, or the non-protein-coding RNA products where applicable, are summarized in the Gene Nomenclature table.

## 3. Results

A total of 914 records were identified through database searching. After removing 209 duplicate records, 705 records remained for screening. Of these, 363 records were excluded based on the predefined exclusion criteria. The remaining 342 records were assessed for eligibility, and 186 were excluded because they did not meet the inclusion criteria. Ultimately, 156 studies were included in the systematic review.

Among the 156 studies included in the systematic review, 106 (67.9%) contained an in vitro component, 67 (42.9%) incorporated an animal (in vivo) model, and 98 (62.8%) included a human clinical study with a case–control or cohort design. Because these categories were not mutually exclusive, several publications combined two or more study-design components, reflecting the heterogeneous and translational nature of the available evidence, ranging from mechanistic laboratory investigations to preclinical models and clinical observations (Supplementary Table S5).

### 3.1. Deep Venous Thrombosis

We identified 99 studies investigating the role of miRNAs in the DVT setting. DVT-associated miRNAs were classified as pro-thrombotic, anti-thrombotic, or unclassified miRNAs. Detailed overviews are presented in Table 1 and Table 2.

### 3.2. Pro-Thrombotic miRNAs in Deep Venous Thrombosis

Thirty studies reported miRNAs with pro-thrombotic properties in the DVT setting. These miRNAs are listed in Table 1.

Elevated miR-92a-3p levels were reported to promote angiogenesis through a *TUG1*/miR-92a-3p/*Hmgcr* axis in DVT; *TUG1* overexpression or miR-92a-3p inhibition reduced thrombosis in vivo [32]. miR-93-5p was shown to regulate endothelial and macrophage functions in DVT. Knockdown of the long noncoding RNA (lncRNA) *NORAD* enhanced human umbilical vein endothelial cell (HUVEC) proliferation and reduced apoptosis and inflammation, whereas miR-93-5p overexpression reversed these effects. In addition, *NORAD* discriminated DVT from controls (AUC 0.919) and predicted PTS [33]. A second study showed that lncRNF219-3:1 sponges miR-93-5p to upregulate T-cell immunoglobulin and mucin domain-containing protein 4, thereby suppressing nuclear factor kappa B (NF-κB)-mediated inflammation [34]. miR-96 was shown to suppress *VAMP8* and reduce platelet activation. Postoperative platelet miR-96 levels correlated with D-dimer levels in orthopedic patients with DVT [35].

miR-125a-5p was reported to be upregulated in patients with DVT, and its levels showed a high diagnostic performance (AUC >0.8 alone; >0.97 combined with D-dimer) for DVT diagnosis. It inhibited endothelial progenitor cell (EPC) migration and angiogenesis through the *MCL1* apoptosis regulator protein and phosphoinositide 3-kinase/protein kinase B (PI3K/Akt) signaling by targeting *ANGPT2* [36,37,38]. miR-128-3p was also upregulated in patients with DVT and promoted endothelial injury by targeting *SIRT1* [39]. Resveratrol-mediated suppression of miR-138 facilitated EPC-mediated DVT recanalization through upregulation of focal adhesion kinase [40]. The *CASC2*/miR-152-3p axis showed high diagnostic performance in a study of 150 patients with DVT and controls; miR-152-3p was reported to target genes involved in transforming growth factor beta and PI3K/Akt signaling [41].

miR-195 was evaluated in four studies and was consistently elevated in patients with DVT. Reported diagnostic performance was very high, with AUC values of up to 1.000. Mechanistic studies identified *GABARAPL1*, *NOS3*, and *BCL2* as targets involved in endothelial function and cell-survival pathways [42,43,44,45]. miR-197-3p was reported to reduce *CXCR2* levels, with downstream upregulation of cyclooxygenase-2 and activation of NF-κB, resulting in reduced endothelial-cell viability and angiogenesis and increased inflammatory cytokine release in DVT patients [46]. miR-199b-5p was increased in patients with DVT after total knee arthroplasty, and the combination of miR-199b-5p and nitric oxide achieved an AUC of 0.847 for DVT diagnosis [47].

miR-206 overexpression inhibited EPC function through *GJA1* upregulation and aggravated thrombosis in mouse models [48]. miR-320 levels were elevated in patients with DVT (fold change 1.58–1.79; AUC 0.70–0.79) and correlated with D-dimer levels. Administration of a miR-320a antagonist reduced thrombus size and prolonged clotting time in a rat DVT model [49,50,51]. miR-374a-5p was elevated in patients with DVT after total hip arthroplasty (AUC up to 0.974). Administration of a miR-374b-5p agonist aggravated DVT formation by decreasing interleukin-10 expression in a mouse model [52,53].

miR-383-5p overexpression increased endothelial-cell apoptosis through the *MALAT1*/miR-383-5p/*BCL2L11* axis [54]. miR-424-5p was evaluated in two DVT studies and was upregulated in patients with DVT, with modest diagnostic performance (AUC 0.630). miR-424-5p was reported to suppress *SNHG12* expression, a change associated with endothelial-cell activation and the release of inflammatory cytokines and adhesion factors, primarily through PI3K/Akt signaling [55,56]. miR-448 was increased in peripheral blood mononuclear cells (PBMCs) from patients with DVT compared with non-DVT controls and contributed to the inflammatory response through downregulation of *SIRT1* [57].

miR-499 levels decreased following curcumin administration in vitro. miR-499 overexpression inhibited the proangiogenic effects of curcumin on endothelial cells by targeting *PTEN* [58]. miR-503-5p was identified as a risk factor for DVT in patients with multiple myeloma (MM) and showed considerable diagnostic performance for DVT prediction (AUC 0.848). miR-503-5p mediated proinflammatory and prothrombotic endothelial responses by targeting *WNT3A* [59]. miR-542-3p inhibited angiogenesis by targeting *ANGPT2* [60], whereas miR-6132 upregulation promoted inflammation by targeting *FOXP3*, resulting in DVT formation [61].

### 3.3. Anti-Thrombotic miRNAs in Deep Venous Thrombosis

Sixty-three studies reported antithrombotic miRNAs in the DVT setting. These miRNAs are listed in Table 2.

The increased levels of let-7e-5p enhanced EPC migration and tube formation by targeting *FASLG*, thereby promoting thrombus revascularization in vivo [62]. miR-9 enhanced EPC migration and angiogenesis through *FGF5* regulation and *TRPM7*/PI3K/Akt/autophagy signaling [63,64]. miR-21 was evaluated in four studies in the DVT setting. Cancer-derived exosomal miR-21 enhanced EPC function through *IL6R* targeting [65]. Reduced miR-21 levels in patients with DVT predicted recurrence and PTS [66]. Berberine improved EPC function by increasing miR-21-3p levels; miR-21-3p targets *RRAGB*, which regulates mechanistic target of rapamycin (mTOR) signaling [67]. The *GAS5*/miR-21/*PTEN* axis suppressed PI3K/Akt/NF-κB signaling, resulting in DVT improvement [68].

miR-22-3p was upregulated in EPCs from DVT patients, and its inhibition enhanced EPC proliferation through the OC1/*VEGFA* pathway. Lower miR-22-3p levels were associated with a reduced risk of DVT recurrence [69]. In another study, the hsa_circ_000455/miR-22-3p/*NLRP3* axis was implicated in DVT [70]. miR-26a was reported to be downregulated in DVT. Its levels correlated inversely with *CCL2* and *CCL7* and showed moderate diagnostic value for DVT (AUC 0.73) [71,72].

The circular RNA circ_0001020 sponged miR-29c-3p and upregulated *MDM2*, thereby impairing EPC function. Knockdown of circ_0001020 reduced thrombosis in a mouse DVT model [73]. miR-103a-3p was reported to be downregulated in DVT in three studies and targeted *CXCL12*, *HMGB1*, and *PTEN* to reduce inflammation and improve EPC function [74,75,76]. miR-122 overexpression protected HUVECs from oxidative stress by suppressing p53 [77].

The *LINC01123*/miR-125a-3p/*IL1R1* axis was shown to promote DVT. Within this axis, miR-125a-3p suppression reduced thrombus burden in a rat DVT model [78]. miR-126 supported EPC function and thrombus resolution by targeting *PIK3R2* and *FOXO4* across three studies [79,80,81]. miR-136-5p was downregulated in DVT, and restoration of its expression reduced thrombus formation and levels of inflammatory mediators, including interleukin-6 (IL-6) and C-reactive protein. EPC-derived extracellular vesicles restored miR-136-5p in a mouse DVT model while suppressing *TXNIP* as a target [82,83].

A circular RNA–miRNA network study identified a CBT15_circR_28491–miR-139-3p–*Kif18a*/*Cdca8*/*Nek2* axis as a potential regulatory pathway in DVT [84]. miR-143-3p promoted EPC function through *ATG2B* regulation and contributed to DVT recanalization. *LINC00659* and *UXT-AS1* were reported to sponge miR-143 in high-altitude DVT and thereby regulate prothrombotic gene expression [85,86]. miR-145 reduced TF expression and thrombogenesis in preclinical DVT models; however, its clinical evaluation in 360 patients with suspected DVT showed low diagnostic performance (AUC 0.59) [87,88]. The circUSP9X/miR-148b-3p/*SRCIN1* axis was implicated in endothelial injury in DVT [89].

miR-150 was reported to be downregulated in EPCs from patients with DVT. Its overexpression enhanced EPC proliferation, angiogenesis, and thrombus resolution through *MYB* and *SRCIN1* in three studies [90,91,92]. The *CRNDE*/miR-181a-5p/*PCYOX1L* axis contributed to thrombus development in a mouse DVT model [93]. miR-181b was reduced in patients with DVT and showed high diagnostic accuracy (AUC 0.880–0.953 when combined with D-dimer). Its overexpression enhanced HUVEC viability and migration through *HEY2* targeting [94,95]. miR-181c-5p overexpression reduced apoptosis, oxidative stress, and thrombosis-related markers in the DVT setting by targeting *FOS* [96].

miR-185 overexpression suppressed TF and fibrin formation by modulating PI3K/Akt and mitogen-activated protein kinase (MAPK) signaling [97]. miR-199a overexpression enhanced EPC-mediated recanalization through *TAB2* targeting [98]. miR-204-5p exerted antithrombotic and anti-inflammatory effects through *SPRED1* and *P4HB* in two DVT studies [99,100]. miR-205 overexpression promoted EPC homing and thrombus recanalization by targeting *PTEN* [101]. The *NEAT1*/miR-218-5p/*GAB2* axis promoted endothelial apoptosis and inflammation in DVT [102].

miR-223 was evaluated in four studies. Its upregulation reduced blood viscosity, decreased inflammatory mediators, and enhanced EPC function. These effects were attributed to regulation of *TLR2*, *MYD88*, c-Jun N-terminal kinase, *FOXO1*, and *NLRP3*. miR-223 also showed high diagnostic value (AUC >0.8) when combined with miR-125a-5p for DVT diagnosis [36,103,104,105]. miR-296-5p regulated endothelial function in DVT by promoting endothelial apoptosis, oxidative stress, and endothelial-to-mesenchymal transition. These effects involved *MEG8* and *S100A4*. Inhibition of miR-296-5p aggravated thrombosis in vivo [106,107].

miR-338-5p directly targeted IL-6, and its overexpression reduced IL-6 levels and attenuated DVT in vivo [108]. Exosome-delivered miR-342-3p inhibited thrombus formation by targeting *EDNRA* [109]. miR-361-5p downregulation accelerated thrombus resolution through *FGF1* targeting [110]. miR-411 overexpression suppressed vein-wall fibrosis in DVT rats through hypoxia-inducible factor-1 alpha-dependent matrix metalloproteinase-2 downregulation [111].

miR-421 directly targets *SERPINE1* mRNA, which encodes PAI-1, thereby influencing fibrinolytic balance [112]. Elevated plasma miR-423-5p levels outperformed D-dimer for the detection of DVT in elderly patients with fractures (AUC 0.663 versus 0.491) [113]. Inhibition of miR-483-3p enhanced EPC homing [114], whereas miR-483-5p-enriched exosomes reduced TF expression and *NLRP3* inflammasome activation by targeting *MAPK1* [115].

miR-495 overexpression suppressed the *IL1R1*/*TLR4* axis and reduced thrombus burden in a rat DVT model [116]. miR-497-5p promoted EPC function and reduced thrombus formation through *LITAF* targeting [117]. miR-513c-5p overexpression reduced caspase-1 levels and attenuated DVT by limiting inflammasome-driven interleukin-1 beta and interleukin-18 production [118]. miR-525-5p enhanced HUVEC viability and reduced apoptosis through the *LINC00659*/miR-525-5p/*BAX* axis in the DVT setting [119].

The role of miR-758-5p in DVT was associated with a single-nucleotide polymorphism (SNP) that altered miR-758-5p binding to *BDKRB2* mRNA, resulting in changes in vascular gene regulation [120]. miR-2467-3p negatively regulated *ABLIM1*, and its knockdown improved endothelial-cell function and reduced inflammation in DVT [121].

miR-3120-5p inhibited the PI3K/Akt/mTOR pathway and matrix metalloproteinase-1 expression, thereby regulating EPC migration and angiogenesis through *WTAPP1* [122]. miR-5189-3p mimics promoted thrombus organization and recanalization through Notch signaling involving *JAG1*, *NOTCH1*, and *HES1* in a rat DVT model [123].

### 3.4. Unclassified miRNAs in Deep Venous Thrombosis

In six studies, we were unable to classify the miRNAs according to their reported roles. One study identified eight differentially expressed miRNAs (miR-150-5p, miR-326, miR-144-3p, miR-199a-5p, miR-199b-5p, miR-125a-5p, let-7e-5p, and miR-381-3p) in human DVT, with enrichment of p53, PI3K/Akt, and MAPK pathways and diagnostic value reported for *PDE1B* and *IRS1* [124]. A stasis-induced rat DVT microarray study identified 19 miRNA regulatory networks enriched in endothelial-cell functional pathways [125]. A mouse inferior vena cava stasis model identified a competing endogenous RNA (ceRNA) network comprising 20 lncRNAs, 54 miRNAs, and 166 mRNAs, with miR-6396, miR-1946a, and miR-466 family members as key nodes [126].

In another study, elevated hsa_circ_101209 in pregnancy-related DVT correlated with D-dimer (r = 0.358) and was predicted by Encyclopedia of RNA Interactomes analyses to bind 15 miRNAs. A three-marker panel achieved an AUC of 0.953 for DVT diagnosis [127]. A multi-omics DVT study identified a hsa_circ_0095124/hsa-miR-3074-5p/*TNFSF13B* ceRNA axis and nominated *JAK2*, *CD36*, *TNFSF13B*, *TLR7*, and *PARP9* as hub regulators [128]. Finally, a study of hip fracture-associated DVT identified a ceRNA network including hsa-miR-93-5p and hsa-miR-320b; the *HSF2*–hsa-miR-139-5p mRNA–miRNA interaction was confirmed by qRT-PCR, linking immune signaling to thrombosis [129].

### 3.5. Pulmonary Embolism

Twenty-five studies investigated the role of miRNAs in the setting of PE, using cell lines, animal models, and clinical samples. PE-associated miRNAs were categorized as prothrombotic or antithrombotic, with unclassified miRNAs summarized separately. Detailed overviews are presented in Table 3 and Table 4.

### 3.6. Prothrombotic miRNAs in Pulmonary Embolism

Fifteen studies reported prothrombotic miRNAs in the PE setting. These miRNAs are listed in Table 3.

let-7b-5p was upregulated in acute PE and aggravated endoplasmic reticulum stress through *SERP1* upregulation [130]. It also suppressed platelet-derived growth factor-stimulated pulmonary artery smooth muscle cell (PASMC) proliferation by targeting *IGF1* [131]. Plasma miRNA profiling identified let-7i-5p, which was upregulated in acute PE and idiopathic pulmonary arterial hypertension (IPAH), and miR-320a, which was elevated in CTEPH and IPAH. *AR* and *PRKCA* were identified as specific targets [132].

In an experimental rat model of acute PE, miR-21 regulated inflammatory responses and lung injury by targeting *PTEN*. Curcumin suppressed this axis and reduced mean pulmonary arterial pressure, right ventricular systolic pressure, and thrombus volume [133]. In another study, berberine suppressed miR-22-5p/*TLR4*/NF-κB signaling in acute PE, thereby reducing PASMC proliferation and pulmonary hypertension [134].

Both miR-27a and miR-27b were elevated in acute PE and showed enhanced diagnostic accuracy when combined with D-dimer (AUC 0.909) [135]. miR-28-3p was elevated in a canine PE model (AUC 0.792) [136] but was reduced in PE endothelial cells, in which its target gene *API5* overexpression protected endothelial cells from apoptosis [137].

Plasma miR-134 was markedly elevated in patients with acute PE and showed strong diagnostic accuracy (AUC 0.833) [138]. miR-190 and miR-197 were also elevated in patients with PE (AUC 0.78 and 0.79, respectively), and their combination with D-dimer achieved an AUC of 0.954 [139]. miR-221 was upregulated in patients with acute PE and showed high diagnostic performance (AUC 0.823). Its levels correlated positively with B-type natriuretic peptide, troponin I, and D-dimer [140]. miR-221-3p promoted PASMC proliferation by targeting *PTEN* in PE-associated pulmonary hypertension [141].

miR-514a-5p overexpression exacerbated right ventricular hypertrophy and lung injury in a rat PE model through *CHRDL1* downregulation [142]. miR-1233 showed high diagnostic performance for acute PE versus non-ST-elevation myocardial infarction (AUC 0.95; 90% sensitivity and 100% specificity) [143]. It also outperformed D-dimer and the Wells score in PE complicating an acute exacerbation of chronic obstructive pulmonary disease (AUC 0.884) [144].

### 3.7. Antithrombotic miRNAs in Pulmonary Embolism

Eight studies reported antithrombotic miRNAs in the PE setting. These miRNAs are listed in Table 4.

miR-22-3p was downregulated in PE-associated cardiac injury. The lncRNA *MALAT1* was reported to sponge miR-22-3p and thereby promote *NLRP3*-mediated inflammasome injury in PE. Resveratrol restored miR-22-3p levels and attenuated PE-induced *NLRP3* activation [145]. miR-34a-3p was downregulated in acute PE, and restoration of its expression reduced mean pulmonary arterial pressure and pulmonary arterial wall thickening through *DUSP1* suppression in PASMCs [146]. The *MEG3*/miR-34a-3p/*DUSP1* axis was subsequently identified as a key regulator of smooth-muscle proliferation and vascular remodeling in PE.

miR-106b-5p downregulation coincided with NOR-1 upregulation in PE models. AgomiR-106b-5p improved survival and attenuated pulmonary vascular proliferation by targeting *NOR-1* [147]. miR-124 regulated endothelial metabolic reprogramming by targeting *PTBP1* in a rat PE model [148]. miR-145 was downregulated in chronic obstructive pulmonary disease-associated PE and correlated positively with the ratio of forced expiratory volume in 1 s to forced vital capacity. Curcumin upregulated miR-145-5p, which targeted *IRS1* and reduced myocardial injury [149,150]. miR-150-5p was reduced in acute PE compared with DVT, with a further reduction in PE complicated by pulmonary arterial hypertension (AUC 0.912 for distinguishing PE from DVT) [151].

### 3.8. Unclassified miRNAs in Pulmonary Embolism

In two studies, we were unable to classify the miRNAs according to their reported roles.

A six-miRNA panel (miR-363-3p, miR-93-3p, miR-22-5p, miR-451a, miR-222-3p, and miR-140-3p) predicted postsurgical PE in patients with glioma (AUC 0.78) and outperformed the Khorana score [152].

In a hypoxia-reoxygenation PE model, urokinase restored dysregulated miRNA patterns, including miR-34a-5p, miR-324-5p, miR-331-3p, miR-429, miR-491-5p, and miR-449a. miR-449a overexpression abolished the protective effects of urokinase, identifying it as a mediator of ischemic injury [153].

### 3.9. Venous Thromboembolism

Thirty-two studies examined miRNA involvement in the VTE setting. Detailed overviews of prothrombotic and antithrombotic miRNAs in VTE are provided in Table 5 and Table 6. The unclassified miRNAs were summarized separately.

### 3.10. Prothrombotic miRNAs in Venous Thromboembolism

Nine studies reported prothrombotic miRNAs in the VTE setting. These miRNAs are listed in Table 5.

Elevated baseline miR-126 levels correlated with residual thrombosis and may predict persistent thrombotic risk in VTE [154]. In a bioinformatic analysis enriched for 16 pathways, miR-130a was predicted to have 225 gene targets [155]. miR-144 levels were increased in VTE and were associated with reduced *FOS* expression in neutrophils, linking adipokine and inflammatory signaling to VTE pathogenesis [156].

In hibernating black bears, miR-141-3p, miR-200a-3p, and miR-200c-3p were downregulated and were predicted to target *SERPINC1*, which encodes antithrombin, suggesting that reduced expression of these miRNAs may lower thrombotic risk [157]. miR-200c-3p was upregulated in rat VTE models, and its inhibition reduced thrombus formation. miR-200c-3p levels also correlated inversely with *SERPINC1* expression across animal and human samples [158].

In a glioma case–control study, miR-221-3p and miR-451a were identified as VTE-associated miRNAs, with platelet activity linked to the miRNA profiles [159]. miR-483-5p levels were increased in VTE and correlated with body mass index and C-reactive protein. miR-483-5p also showed independent predictive value for VTE diagnosis, particularly in patients with lung cancer and a high body mass index [160].

Plasma miR-3613-5p showed high diagnostic accuracy (AUC 0.805) among multiple upregulated miRNAs in acute VTE and was functionally linked to decreased fibrinogen and increased platelet aggregation [161]. In another study, six miRNAs (miR-4651, miR-4734, miR-6798-3p, miR-6789-5p, miR-6765-5p, and miR-6816-5p) showed at least fivefold increases in VTE. Pathway analyses implicated thrombomodulin and platelet-related gene regulation as potential targets of these miRNAs [162].

### 3.11. Antithrombotic miRNAs in Venous Thromboembolism

Seven studies reported antithrombotic miRNAs in the VTE setting. These miRNAs are listed in Table 6.

The lncRNA *ANRIL* promoted angiogenesis and thrombosis by modulating miR-99a and miR-449a in the autophagy pathway in a rat VTE model [163]. Estradiol-responsive miR-365a-3p targeted the tissue factor 3′ untranslated region and reduced TF-initiated thrombin generation. miR-365a-3p was also downregulated in pregnant women and oral contraceptive users [164]. miR-200a-5p was downregulated in patients with VTE, and its overexpression enhanced endothelial-cell migration by targeting *COX7C* [165].

In patients with cervical cancer, abnormal expression of miR-195-5p, miR-205-5p, and vascular endothelial growth factor A was associated with VTE treatment outcomes [166]. Circulating miR-522-3p and miR-134 were upregulated in patients with VTE, and combined miRNA and gene-expression profiling supported their diagnostic potential [167].

In a prospective population-based study (the HUNT study), individuals in the highest miR-145 quartile had a 49% lower risk of VTE (hazard ratio 0.51), with a stronger association for unprovoked VTE, supporting miR-145 as an inverse VTE risk biomarker [168]. In cervical cancer-related VTE, lower miR-20a-5p levels predicted VTE, whereas lower miR-145-5p levels correlated with improved survival in patients with cervical adenocarcinoma [169].

### 3.12. Unclassified miRNAs in Venous Thromboembolism

In sixteen studies, we were unable to classify the miRNAs according to their reported roles.

SNPs in miR-133a2 were associated with warfarin dose variability. GA or AA genotypes required higher weekly doses than wild-type genotypes, supporting a potential role for miR-133a2 polymorphisms in personalized anticoagulation [170]. Profiling of 97 plasma miRNAs identified distinct signatures that separated patients with VTE from controls, with miR-10b-5p, miR-320a/b, miR-424-5p, and miR-423-5p upregulated and miR-103a-3p, miR-191-5p, miR-301a-3p, and miR-199b-3p downregulated [171].

In a VTE cohort, 12 miRNAs were significantly associated with recurrence; miR-15b-5p, miR-197-3p, miR-27b-3p, and miR-30c-5p showed time-dependent trends preceding earlier recurrences, and eight miRNAs correlated with transforming growth factor beta 1/2 signaling [172]. Combined polymorphism analyses in Korean patients with VTE identified three allelic combinations of miR-146a, miR-149, miR-196a2, and miR-499 that were significantly associated with increased VTE risk, supporting polygenic susceptibility [173].

*RAN* rs14035 and *XPO5* rs11077 polymorphisms were associated with VTE prevalence, and haplotype analysis across *DICER1*, *DROSHA*, *RAN*, and *XPO5* identified protective and risk-conferring variants [174]. A Bayesian network analysis linked miR-199b-3p to hematocrit and VTE risk; miR-370, miR-27b-3p, and miR-222-3p were associated with recurrence; and 21 novel SNP-miRNA associations were identified in a genome-wide association study [175].

Four circulating miRNAs (miR-126-3p, miR-885-5p, miR-194-5p, and miR-192-5p) showed significant associations with VTE (odds ratios 1.3–1.8), and a combined risk model including age and sex achieved an AUC of 0.77 [176]. A seven-miRNA signature predicted future VTE risk (AUC 0.95) and was enriched in complement and coagulation-cascade pathways; adding calprotectin improved risk stratification (AUC 0.77) [177].

Large-scale sequencing and bioinformatic studies further characterized the miRNA regulatory landscape in VTE: 547 dysregulated miRNAs were identified in one study [178], and 32 differentially expressed miRNAs implicated hemostatic pathways in another [179]. A multicohort analysis of 1950 miRNAs identified a weighted miRNA score that predicted VTE outcomes over 8 years and delineated five distinct miRNA networks involving fibrinolytic signaling [180]. An integrative analysis identified 89 dysregulated miRNAs and *CREB1* as a central regulator and validated *ESM1*, *HIF1A*, and *CREB1* in unprovoked VTE [181].

A bioinformatic study of the thrombin-activatable fibrinolysis inhibitor interactome identified 28 hemostasis-related genes targeted by 24 miRNAs that were enriched in complement/coagulation cascades and neutrophil extracellular-trap formation [182]. A comparative analysis across thrombotic and inflammatory disorders identified 30 shared dysregulated miRNAs linking immunothrombosis and epigenetic regulation [183].

In coronavirus disease 2019-related VTE, 42 shared differentially expressed genes were identified, with eight hub genes, including *COX7C*, *RSL24D1*, and *NDUFA4*, nominated as diagnostic markers within dense transcription-factor–gene and transcription-factor–miRNA co-regulatory networks [184]. A separate study examined lymphangiogenesis-related VTE mechanisms: *MYC*, regulated by hsa-miR-449c-5p, and *JUN* were identified as central hubs, and qRT-PCR confirmed *MYC* downregulation and *NTAN1* upregulation in VTE, linking immune-metabolic dysregulation to thrombotic progression [185].

## 4. Discussion

This systematic review synthesized 156 studies on miRNAs in VTE pathogenesis: 99 on DVT, 25 on PE, and 32 on VTE. Several miRNAs were consistently reported across multiple studies, underscoring their potential as biomarkers or therapeutic targets. Among prothrombotic DVT miRNAs, miR-195, miR-125a-5p, miR-320, miR-93-5p, miR-374, and miR-424-5p were validated in both functional and clinical settings. Antithrombotic DVT miRNAs confirmed in multiple studies included miR-21, miR-223, miR-103a-3p, miR-126, miR-150, miR-9, miR-22-3p, miR-26a, miR-136-5p, miR-143, miR-145, miR-181b, miR-204-5p, and miR-296-5p. In PE, prothrombotic miRNAs including let-7b-5p, miR-28-3p, miR-221, and miR-1233 were reproducibly identified, while miR-134 and miR-190/miR-197 showed particularly notable diagnostic performance. Antithrombotic miR-34a-3p and miR-145 were evaluated in multiple PE studies, with miR-145 and miR-150 demonstrating noteworthy cardiopulmonary protective effects.

**Figure 2:**
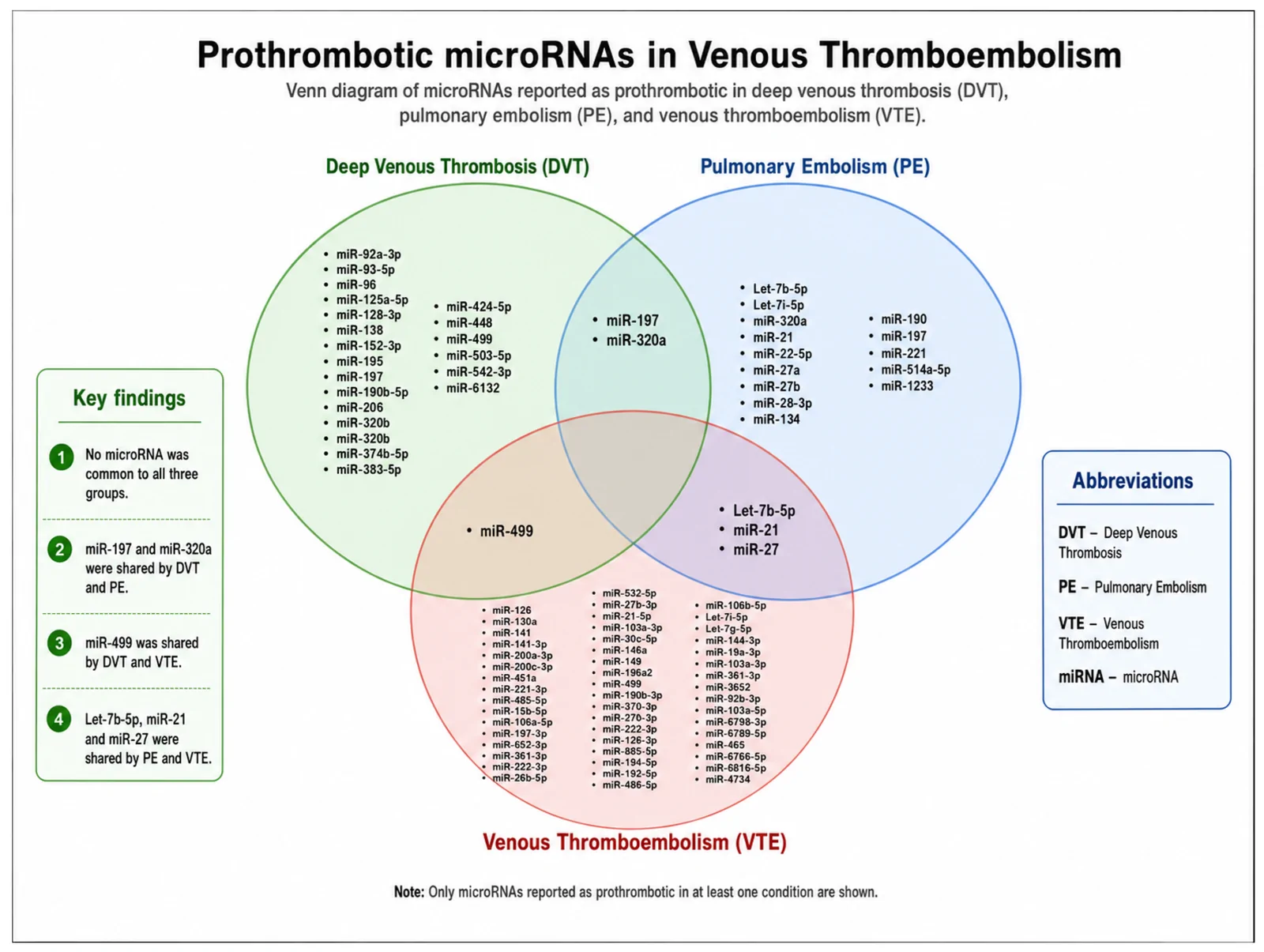
Overlap of prothrombotic miRNAs across venous thromboembolism (VTE) entities. Across deep vein thrombosis (DVT), pulmonary embolism (PE), and overall VTE cohorts, no single prothrombotic miRNA was shared among all three conditions. Partial overlaps were observed between disease entities: let-7b-5p, let-7i-5p, miR-21, and miR-27 were shared between PE and VTE; miR-197-3p and miR-499 were shared between DVT and VTE; and miR-197 and miR-320a were shared between DVT and PE. These findings highlight both shared and condition-specific miRNA signatures across the VTE spectrum.

**Figure 3:**
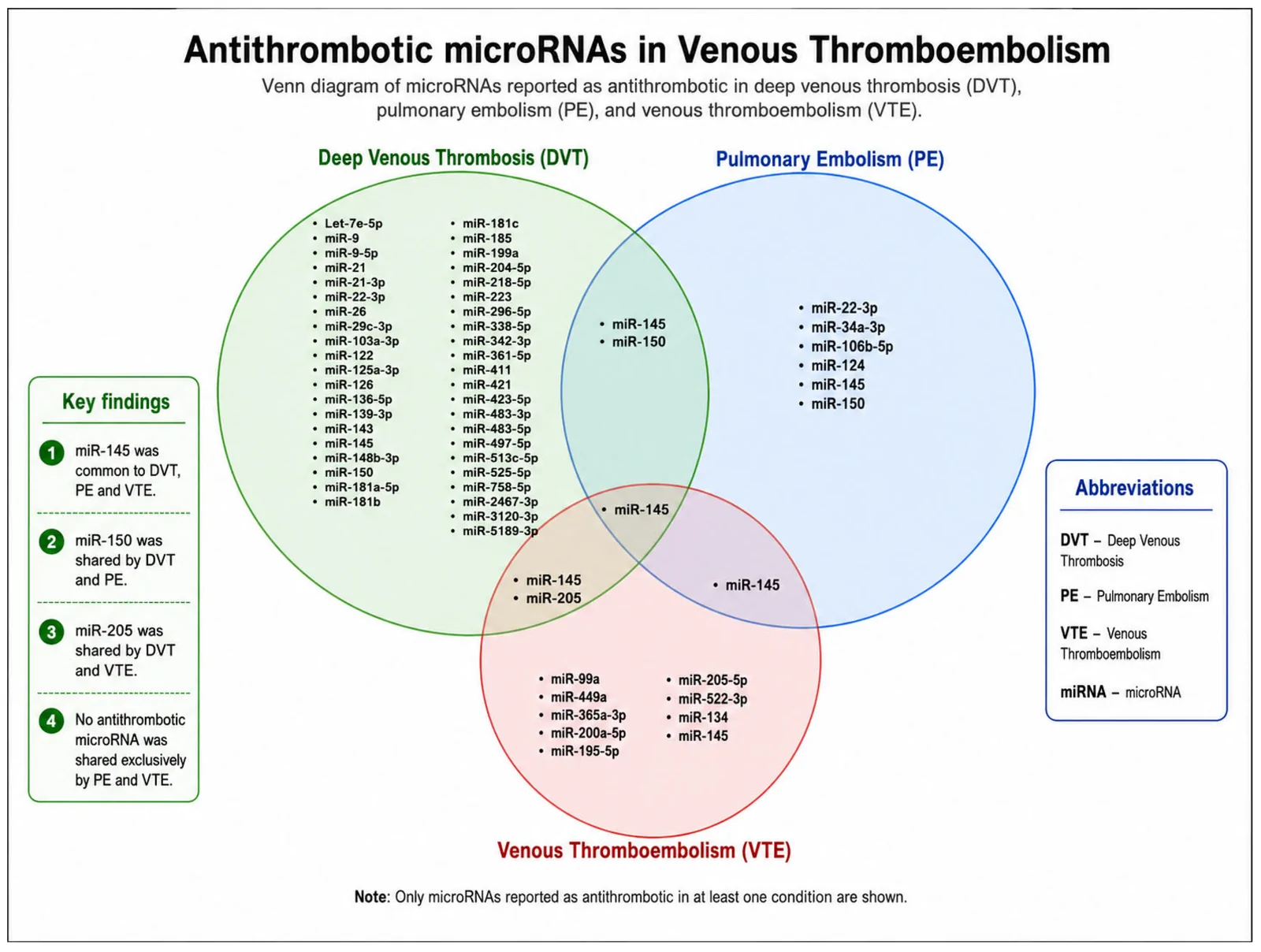
Overlap of antithrombotic miRNAs across venous thromboembolism (VTE) entities. In contrast to the prothrombotic profile, miR-145 was the only antithrombotic miRNA identified across deep vein thrombosis (DVT), pulmonary embolism (PE), and overall VTE cohorts. Additional partial overlaps were observed: miR-150 was shared between DVT and PE, and miR-205 was shared between DVT and VTE, indicating a more limited intersection of antithrombotic miRNA signatures across VTE phenotypes.

Across the three VTE categories, no single prothrombotic miRNA was shared among DVT, PE, and VTE simultaneously. let-7b-5p, let-7i-5p, miR-21, and miR-27 were shared between PE and VTE; miR-197-3p and miR-499 were shared between DVT and VTE; and miR-197 and miR-320a were shared between DVT and PE (Figure 2). In contrast, miR-145 was the only antithrombotic miRNA identified across all three categories; miR-150 was shared between DVT and PE, and miR-205 between DVT and VTE (Figure 3). These patterns suggest that, although DVT, PE, and VTE share overlapping molecular pathways, many miRNAs may have condition-specific rather than universally applicable biomarker potential.

In our systematic review, the classification of miRNAs as prothrombotic or antithrombotic was based on the net reported effect on thrombus formation or burden in each source study, rather than on a single, direct molecular mechanism. This binary classification enabled us to pragmatically organize the reported miRNAs by simplifying a more complex biology. Many of the miRNAs discussed in this manuscript act primarily on endothelial repair, inflammation, angiogenesis, apoptosis, or immune (immunothrombotic) signaling rather than on the coagulation cascade itself. Furthermore, several miRNAs exhibit pleiotropic, context-dependent, and occasionally opposing effects across different experimental settings or VTE entities. The “unclassified” category reflects miRNAs for which the available evidence did not support a consistent net direction. These labels should therefore be read as descriptive summaries of predominant reported effects, not as fixed intrinsic properties.

Previous literature reviews and metanalyses have consistently described the circulating miRNAs as promising but not yet clinically validated biomarkers because most studies are small, heterogeneous, and lack standardized sampling, normalization, and external validation. These limitations make it difficult to determine whether altered miRNA levels contribute causally to thrombosis or merely reflect the thrombotic event [186]. Our systematic review extends these earlier reviews by evaluating 156 publications, separating evidence across DVT, PE, and VTE settings, and distinguishing clinical observations from functional and preclinical findings. The limited overlap of individual miRNAs across these entities supports the view that single miRNAs are unlikely to provide universally applicable biomarkers. Previous reviews have similarly proposed combining miRNA signatures with conventional biomarkers and incorporating them into existing VTE and cancer-associated thrombosis risk models [26,187]. These reviews also reinforce our pathway-based interpretation [188,189]. Related to this, cancer-associated thrombosis deserves particular consideration because tumor-derived factors, anticancer treatment, inflammation, and tissue-factor signaling create a distinct thrombotic environment [190].

In our systematic review, several miRNAs converged on shared molecular targets: ANGPT2 was targeted by prothrombotic miR-125a-5p and miR-542-3p; SIRT1 was regulated by miR-128-3p and miR-410-3p; and PTEN was influenced by both prothrombotic miRNAs (miR-499, miR-21, miR-221) and the antithrombotic miR-205. Some miRNAs demonstrated opposing effects across VTE contexts, highlighting the complex and context-dependent regulatory roles of miRNAs in thrombosis. Most miRNAs were examined in single studies with considerable methodological heterogeneity. A significant proportion relied on animal models, raising concerns about translatability to human VTE pathophysiology. Although many miRNAs are generally highly conserved across diverse species and share similar functions in target gene regulation, many miRNAs may not be conserved between distantly related species or even closely related species depending on the specific miRNA and species pair involved [191]. The conserved miRNAs do not necessarily exhibit the same expression levels or patterns in different species or at different stages within a species.

The identification of consistently significant miRNAs across multiple studies suggests diagnostic, prognostic, and therapeutic implications, particularly for prothrombotic miRNAs such as let-7b-5p, miR-28-3p, miR-125a-5p, miR-134, miR-190/miR-197, miR-195, miR-221, miR-320, and miR-1233, and antithrombotic miRNAs such as miR-26a, miR-34a-3p, miR-181b, miR-223, miR-145, and miR-150. Future research should prioritize human-specific models, standardized methodologies, and large-scale validation studies.

Across the reviewed literature, individual miRNAs converge on a limited set of recurring molecular pathways, which we summarize here rather than reiterating study-by-study findings. First, PI3K/Akt (and downstream mTOR) signaling recurs as a hub for endothelial and endothelial-progenitor-cell proliferation, migration, and thrombus recanalization, engaged by numerous miRNAs on both sides of the classification (e.g., via PTEN, a shared node targeted by prothrombotic miR-21/miR-221/miR-499 and antithrombotic miR-205). Second, NF-κB–driven inflammation and NLRP3 inflammasome activation define an immune-thrombosis axis linking endothelial injury, leukocyte activation, and cytokine release (e.g., miR-223, miR-448, miR-513c-5p, miR-22-3p). Third, a smaller group acts directly on the coagulation and fibrinolytic machinery such as tissue factor (miR-145, miR-185, miR-365a-3p), PAI-1 (miR-421), and antithrombin/SERPINC1 (miR-200c-3p, miR-141-3p), representing the most mechanistically proximal effects on thrombus formation. Fourth, endothelial repair and angiogenesis, frequently mediated through ANGPT2 and VEGFA, recur across EPC-focused studies. Finally, apoptosis and oxidative-stress pathways (Bcl-2 family, p53) modulate endothelial survival. Recognizing these convergent pathways provides more traceable targets for mechanistic and therapeutic investigation and helps explain the pleiotropy and context-dependence noted above.

The findings should be interpreted according to their level of evidence (Supplementary Tables S2, S3, S4 and S5). The majority of the included studies were preclinical (in vitro and animal), and a large proportion of human studies were single-center case–control designs with limited sample sizes and without independent validation. The results of in vitro studies and animal findings should be regarded as hypothesis-generating, and greater confidence should be reserved for miRNAs in which concordant preclinical evidence is accompanied by validated human data.

The diagnostic estimates summarized here should also be interpreted with caution. Many derive from small, single-center, often retrospective cohorts without independent validation, a setting in which discrimination is systematically overestimated; near-perfect values, such as the reported AUC of 1.000 for miR-195, most likely reflect overfitting or small-sample optimism rather than genuine diagnostic accuracy. Moreover, few studies benchmarked miRNA performance against established diagnostics (D-dimer) or validated clinical probability scores (Wells, Geneva), and fewer still evaluated incremental value when added to these tools. Accordingly, the miRNAs discussed here should be regarded as exploratory biomarker candidates that require prospective, externally validated, and comparative evaluation before any diagnostic application can be considered.

Beyond the strength of the underlying associations, several practical barriers currently preclude the clinical translation of circulating-miRNA diagnostics in VTE. At the pre-analytical level, miRNA measurements are sensitive to the choice of matrix (serum versus plasma), to platelet and cellular contamination, and particularly to hemolysis, which spuriously elevates several red-cell–enriched miRNAs and can confound results if not controlled. At the analytical level, there is no consensus endogenous normalizer for circulating miRNAs, and quantification is platform-dependent (qRT-PCR, microarray, and next-generation sequencing yield non-interchangeable values), so results are frequently not reproducible across laboratories in the absence of standardized, validated assays. At the implementation level, miRNA assays remain more expensive and slower than D-dimer, and more importantly few studies have compared miRNAs against D-dimer or demonstrated their diagnostic value with validated clinical probability–based algorithms (e.g., Wells or Geneva scores). Until assay standardization, normalization, and prospective comparative validation are achieved, miRNAs are best regarded as complementary research tools rather than deployable clinical diagnostics.

## 5. Limitations

Several limitations should be acknowledged. First, the evidence base is dominated by preclinical (in vitro and animal) studies, and miRNA sequences and functions are not fully conserved across species, limiting translatability to human VTE. Second, most human studies were small, single-center, case–control investigations without independent validation cohorts, and the risk-of-bias appraisal (Supplementary Table S2) indicated corresponding methodological concerns; reported diagnostic metrics such as AUCs should therefore be regarded as exploratory. Third, the human studies used heterogeneous biological matrices (serum, plasma, extracellular vesicles, mononuclear cells) and differing RNA-isolation and normalization strategies, which limits cross-study comparability. Fourth, the review was not prospectively registered, and no protocol was published; the search was restricted to PubMed and EMBASE and to English-language publications, which may have introduced selection and language bias, and a positive-result (publication) bias is likely in this literature. Finally, because of the pronounced heterogeneity of study designs and endpoints, a quantitative synthesis was not feasible, and no meta-analysis was performed.

## 6. Conclusions

This systematic review synthesized 156 studies investigating miRNAs in venous thromboembolism (VTE), including 99 studies of deep vein thrombosis (DVT), 25 of pulmonary embolism (PE), and 32 of VTE. The available evidence demonstrates that miRNAs are involved in several key biological pathways relevant to thrombogenesis, particularly endothelial dysfunction, inflammation and immunothrombosis, platelet activation, coagulation cascade activation, impaired fibrinolysis, angiogenesis, and vascular repair. These findings highlight the important role of miRNAs in the pathophysiology of VTE and identify multiple candidates with potential mechanistic and therapeutic relevance. Several circulating miRNAs have also demonstrated promising diagnostic performance in human studies; however, no circulating miRNA can currently be considered a sufficiently validated and reliable clinical biomarker of VTE. Most diagnostic findings are derived from relatively small, single-center studies without independent external validation, while substantial heterogeneity remains in biospecimen selection, RNA isolation, normalization strategies, and analytical platforms. Accordingly, the circulating miRNAs identified in this review should be regarded as promising biomarker candidates rather than established clinical biomarkers. Future research should prioritize adequately powered, prospective, multicenter human studies using standardized analytical approaches, with direct comparisons against D-dimer and established clinical diagnostic algorithms. Integrating functional and mechanistic investigations with well-characterized clinical datasets will be critical to establish the diagnostic, prognostic, and therapeutic utility of miRNA-based approaches and to facilitate their eventual translation into the management of VTE.

## Figures and Tables

**Table 1:** Prothrombotic miRNAs in deep vein thrombosis.

Reference	miRNA	Experimental Model(s)	Human Cohort/Sample	Expression	Principal Reported Effect	Target(s)	Diagnostic Potential
Feng, 2022 [32]	miR-92a-3p	EPCs and a mouse DVT model.	NR	Up	Inhibits angiogenesis.	*TUG1* and *Hmgcr*	No
Zhou, 2024 [33] Wang, 2025 [34]	miR-93-5p	HUVECs and macrophages.	Patients with DVT and controls.	Up	Regulates *NORAD* and suppresses T-cell immunoglobulin and mucin domain-containing protein 4 in macrophages.	*STAT3*, *MAPK1* *TIMD4*	Yes
Xie, 2016 [35]	miR-96	NR	Platelets from patients with DVT after orthopedic surgery.	Up	Suppresses *VAMP8* and reduces platelet activation.	*VAMP8*	Yes
Xu, 2022 [36] Huang, 2023 [37] Yu, 2023 [38]	miR-125a-5p	EPCs and HUVECs.	Serum/plasma samples from patients with DVT.	Up	Suppresses EPC migration and angiogenesis and promotes apoptosis.	*ANGPT2* *MCL1*	Yes (AUC > 0.97)
Han, 2023 [39]	miR-128-3p	HUVECs.	Serum samples from patients with DVT.	Up	Promotes endothelial injury.	*SIRT1*	Yes
Zhang, 2018 [40]	miR-138	EPCs, Murine and mouse DVT models.	NR	Up	Impairs EPC function and thrombus resolution.	*FAK*	No
Wang, 2025 [41]	miR-152-3p	HUVECs.	Serum samples from patients with DVT.	Up	Promotes endothelial injury.	Transforming growth factor beta and PI3K/Akt pathways	Yes
Qin, 2015 [42] Mo, 2016 [43] Qin, 2018 [44] Jin, 2019 [45]	miR-195	EPCs, ECs, and HUVECs.	Plasma samples from patients with DVT.	Up	Modulates EC proliferation, survival, and nitric oxide release.	*GABARAPL1* *NOS3* *BCL2*	Yes (AUC = 1.000)
Tian, 2022 [46]	miR-197-3p	HUVECs.	PBMCs from patients with DVT.	Down	Regulates inflammatory cytokines.	*CXCR2*	No
Zhou, 2024 [47]	miR-199b-5p	NR	Plasma samples from patients undergoing total knee arthroplasty.	Up	NR	NR	Yes (AUC = 0.847)
Li, 2022 [48]	miR-206	A mouse DVT model.	EPCs from DVT patients.	Up	Inhibits EPC function and angiogenesis and promotes apoptosis.	*GJA1*	No
Jiang, 2017 [49] Srivastava, 2019 [50] Srivastava, 2024 [51]	miR-320	A DVT model in SD rats.	Plasma samples from DVT patients.	Up	Promotes thrombus formation and correlates with D-dimer levels.	NR	Yes (AUC = 0.79)
Tang, 2021 [52] Zhang, 2020 [53]	miR-374	A murine DVT model.	PBMCs from elderly patients undergoing hip arthroplasty.	Up	Increases thrombus formation and regulates inflammation.	IL-10	Yes (AUC = 0.974)
Wang, 2022 [54]	miR-383-5p	HUVECs.	NR	Up	Promotes apoptosis.	*BCL2L11*	No
Wang, 2016 [55] Xiao, 2024 [56]	miR-424-5p	HUVECs.	Plasma samples from patients with suspected DVT.	Up	Inhibits EC function and promotes inflammation.	208 overlapping target genes.	Yes
Wang, 2022 [57]	miR-448	HeLa cells.	PBMCs from patients with DVT.	Up	Promotes inflammatory responses.	*SIRT1*	Yes
Wang, 2021 [58]	miR-499	HUVECs and a mouse DVT model.	NR	Up	Inhibits angiogenesis and impairs thrombus resolution.	*PTEN*	No
Guo, 2025 [59]	miR-503-5p	HUVECs cultured with serum from MM patients.	Serum samples from MM patients with and without DVT	Up	Induces endothelial dysfunction.	*WNT3A*	Yes
Lu, 2019 [60]	miR-542-3p	EPCs and a rat DVT model.	NR	Up	Inhibits angiogenesis.	*ANGPT2*	No
Zhang, 2024 [61]	miR-6132	293T cells and a mouse DVT model.	PBMCs from patients with DVT.	Up	Regulates inflammation.	*FOXP3*	Yes

Abbreviations: AUC, area under the receiver operating characteristic curve; DVT, deep vein thrombosis; EC, endothelial cell; EPC, endothelial progenitor cell; HUVEC, human umbilical vein endothelial cell; MM, multiple myeloma; PBMC, peripheral blood mononuclear cell; SD, Sprague-Dawley; NR, not reported. Expression indicates the direction reported in the cited study.

**Table 2:** Antithrombotic miRNAs in deep vein thrombosis.

Reference	miRNA	Experimental Model(s)	Human Cohort/Sample	Expression	Principal Reported Effect	Target(s)	Diagnostic Potential
Kong, 2016 [62]	let-7e-5p	EPCs and a rat DVT model.	NR	Down	Promotes vascular repair and thrombus resolution.	*FASLG*	No
Zhu, 2020 [63] Zhou, 2020 [64]	miR-9 miR-9-5p	EPCs	NR	Up	Enhances EPC migration and angiogenesis.	*FGF5* and *TRPM7*	No
Wang, 2018 [65] Du, 2019 [66] Qiao, 2024 [67] Wang, 2026 [68]	miR-21 miR-21-3p	EPCs, DVT models in mouse and rat.	Samples from patients with venous leg ulcers.	Down	Promotes EPC proliferation, migration, and invasion. Promotes angiogenesis.	*IL6R*, *FASLG*, *RRAGB*	Yes
Liang, 2020 [69] Lou, 2022 [70]	miR-22-3p	EPCs	Blood samples from DVT patients and healthy controls.	Up	Negatively regulates EPC proliferation and migration.	*VEGFA*, *NLRP3*	Yes
Li, 2017 [71] Rahmanpour, 2022 [72]	miR-26	HUVECs	Serum samples from DVT patients and controls.	Down	Inhibits inflammatory pathways.	*PRKCD* *CCL2* and *CCL7*	Yes (AUC = 0.73)
Lou, 2022 [73]	miR-29c-3p	EPCs and a mouse DVT model.	Peripheral blood samples of DVT patients.	Down	Inhibits EPC migration, invasion, and tube formation.	*MDM2*	Yes
Sun, 2020 [74] Cao, 2022 [75] Zhang, 2020 [76]	miR-103a-3p	EPCs, HUVECs, and a mouse DVT model.	Plasma samples from DVT patients.	Down	Inhibits inflammatory pathways and reduces EPC dysfunction.	*CXCL12* *HMGB1* *PTEN*	Yes
Li, 2020 [77]	miR-122	HUVECs.	Serum samples from DVT patients and controls.	Up	Exerts protective effects on endothelial cells.	*TP53*	Yes
Yang, 2022 [78]	miR-125a-3p	EPCs, HUVECs, and a DVT model in SD rats.	NR	Down	Reduces thrombus formation and inflammatory responses.	*IL1R1*	No
Meng, 2015 [79] Chen, 2016 [80] Zhu, 2023 [81]	miR-126	EPCs, VECs, and SD Rat traumatic limb DVT model.	NR	Down	Promotes EPC migration, angiogenesis, and thrombus resolution.	*PIK3R2*	No
Ou, 2020 [82] Feng, 2022 [83]	miR-136-5p	EPCs, HUVECs and DVT models in mice and rats.	Plasma samples from DVT patients and controls.	Down	Reduces inflammation, endothelial-cell apoptosis, and thrombus formation.	IL-6 *TXNIP*	Yes
Chen, 2025 [84]	miR-139-3p	A rat DVT model.	Bioinformatic analysis of human dataset	NR	Is associated with increased expression of prothrombotic cell-cycle-related hub genes.	Predicted hub gene targets: *Kif18a*, *Cdca8*, and *Nek2*	No
Zhang, 2020 [85] Jha, 2021 [86]	miR-143	EPCs.	Plasma samples of DVT patients.	Up	Enhances EPC viability, migration, invasion, and tube formation.	*ATG2B*	No
Sahu, 2017 [87] Antoun, 2024 [88]	miR-145	A DVT model in SD rats.	Plasma samples of DVT patients.	Down	Reduce thrombogenesis by decreasing tissue factor levels.	*TF*	Yes (AUC = 0.59)
Wang, 2024 [89]	miR-148b-3p	A hypoxic HUVEC model and a mouse DVT model.	Blood samples from DVT patients.	Down	Reduces inflammation and endothelial-cell apoptosis and promotes cell viability.	*SRCIN1*	Yes
Wang, 2014 [90] Wang, 2019 [91] Du, 2020 [92]	miR-150	EPCs, DVT model in rats, SD rats and murine model of DVT.	Blood samples from DVT patients and controls.	Down	Enhances EPC migration, angiogenesis, proliferation, and thrombus resolution.	*MYB*, *SRCIN1*	No
He, 2023 [93]	miR-181a-5p	A DVT model in mice.	NR	Down	Reduces inflammation and thrombus formation.	*Pcyox1l*	No
Zhang, 2021 [94] Zhang, 2024 [95]	miR-181b	HUVECs.	Plasma samples from patients with DVT.	Down	NR	*HEY2*	Yes (AUC = 0.953)
Yang, 2023 [96]	miR-181c-5p	HUVECs.	Blood samples from patients with DVT and controls.	Down	Improves cell viability, reduces apoptosis and oxidative stress, and decreases thrombosis-related factors.	*FOS*	Yes
Lu, 2022 [97]	miR-185	Rat deep-vein endothelial cells and a DVT model in SD rats.	NR	Up	Inhibits tissue factor and fibrin formation.	PI3K/AKT and MAPK pathways	No
Ni, 2023 [98]	miR-199a	EPCs and a rat DVT model.	NR	Down	Promotes EPC function and enhances recanalization.	*TAB2*	No
Ding, 2022 [99] Chen, 2023 [100]	miR-204-5p	EPCs, HUVECs, and a rat DVT model.	Plasma samples from patients with DVT and controls.	Down	Improves EPC viability, migration, and angiogenesis.	*SPRED1* *P4HB*	Yes
Sun, 2019 [101]	miR-205	EPCs and a DVT model.	NR	Down	Promotes EPC angiogenesis, migration, invasion, and proliferation and inhibits apoptosis.	*PTEN*	No
Wang, 2023 [102]	miR-218-5p	HUVECs.	Plasma samples from patients with DVT and controls.	Down	Reduces HUVEC apoptosis and inflammation and promotes proliferation and migration.	*GAB2*	Yes
Li, 2020 [103] Sun, 2020 [104] Xu, 2022 [36] Luo, 2025 [105]	miR-223	EPCs and DVT models in mice and rats.	NR	Up	Reduces blood viscosity, lowers inflammatory markers, and enhances EPC function.	*TLR2*, *MYD88*, *c*-Jun N-terminal kinase, *FOXO1* and *NLRP3*	No
Pan, 2021 [106] Zhang, 2025 [107]	miR-296-5p	HUVECs and a mouse DVT model.	Blood samples from DVT patients and controls.	Down	Promotes endothelial apoptosis, oxidative stress, and endothelial-to-mesenchymal transition.	*S100A4*, *MEG8*	Yes
Zhang, 2020 [108]	miR-338-5p	HeLa cells, HUVECs, and a mouse DVT model.	PBMCs from patients with DVT.	Down	Reduces inflammation and DVT formation.	IL-6	Yes
Pan, 2022 [109]	miR-342-3p	HUVECs, human umbilical cord mesenchymal stem cells, and a rat DVT model.	NR	Down	Inhibits thrombus formation and promotes angiogenesis in HUVECs.	*EDNRA*	No
Yang, 2020 [110]	miR-361-5p	EPCs and a DVT model in SD rats.	NR	Down	Enhances EPC proliferation, migration, and tube formation and promotes vascular repair and thrombus dissolution.	*FGF1*	No
Ai, 2018 [111]	miR-411	VSMCs and an injury-induced rat model of DVT-associated vein-wall fibrosis.	NR	Up	Suppresses vascular remodeling and fibrosis.	*HIF-1α*	No
Marchand, 2012 [112]	miR-421	HUVECs.	Plasma samples from DVT patients.	Up	Directly inhibits PAI-1 mRNA translation.	*SERPINE1*	Yes
Chang, 2019 [113]	miR-423-5p	NR	Plasma samples of elderly lower extremity fracture patients with and without DVT.	Up	NR	NR	Yes (AUC = 0.663)
Kong, 2016 [114]	miR-483-3p	EPCs and a rat DVT model.	EPCs isolated from DVT patients and controls.	Down	Reduces inflammation and apoptosis in EPCs; enhances angiogenesis and tissue repair	*SRF*	Yes
Fan, 2024 [115]	miR-483-5p	Adipose stem cell-derived exosomes, HUVECs, and a rat DVT model.	NR	Down	Improves EPC homing, reduces tissue factor expression, and decreases inflammation.	*MAPK1*	No
Tang, 2018 [116]	miR-495	Femoral-vein ECs and a mouse DVT model.	NR	Up	Reduces inflammation and thrombus formation.	*IL1R1*	No
Xu, 2025 [117]	miR-497-5p	EPCs and a rat DVT model.	NR	Down	Promotes EPC proliferation, migration, and angiogenesis and reduces thrombus formation.	*LITAF*	No
Chu, 2022 [118]	miR-513c-5p	HUVECs and a DVT model in mice.	PBMCs from patients with DVT and healthy controls.	Down	Reduces caspase-1 and decreases interleukin-1 beta and interleukin-18 levels.	*CASP1*	Yes
Zhu, 2023 [119]	miR-525-5p	HUVECs	NR.	Up	Enhances HUVEC viability and reduces apoptosis.	*BAX*	No
Wang, 2018 [120]	miR-758-5p	NR	Patients undergoing knee or hip orthopedic surgery, with or without DVT.	Down	A single-nucleotide polymorphism alters *BDKRB2* expression.	*BDKRB2*	Yes
Qui, 2024 [121]	miR-2467-3p	HUVECs and a rat DVT model.	NR	Down	Inhibits inflammation and oxidative stress and reduces thrombus formation.	*ABLIM1*	No
Li, 2018 [122]	miR-3120-5p	EPCs.	NR	Down	Decreases matrix metalloproteinase-1 expression and inhibits the PI3K/Akt/mTOR pathway.	*WTAPP1*	No
Lu, 2021 [123]	miR-5189-3p	A DVT model in SD rats.	Blood samples from DVT patients and controls.	Down	Promotes cell proliferation and reduces apoptosis.	*JAG1*, *NOTCH1*, *HES1*, *BCL2*, *BAX*	Yes

Abbreviations: AUC, area under the receiver operating characteristic curve; DVT, deep vein thrombosis; EC, endothelial cell; EPC, endothelial progenitor cell; HUVEC, human umbilical vein endothelial cell; PBMC, peripheral blood mononuclear cell; SD, Sprague-Dawley; VEC, vascular endothelial cell; VSMC, vascular smooth muscle cell; NR, not reported. Expression indicates the direction reported in the cited study.

**Table 3:** Prothrombotic miRNAs in pulmonary embolism.

Reference	miRNA	Experimental Model(s)	Human Cohort/Sample	Expression	Principal Reported Effect	Target(s)	Diagnostic Potential
Liu, 2020 [130] Zhang, 2022 [131]	let-7b-5p	Human lung epithelial cells (BEAS-2B), PASMCs, and a rabbit PE model.	NR	Up	Modulates endoplasmic reticulum stress and suppresses proliferation and migration of platelet-derived growth factor-induced PASMCs.	*SERP1* *IGF1*	No
Fabro, 2021 [132]	let-7i-5p miR-320a	NR	Plasma samples from patients with acute PE, CTEPH, or IPAH.	Up	Regulates pulmonary arterial adventitial fibroblasts, pulmonary artery endothelial cells, and PASMCs.	*AR* *PRKCA*	Yes
Liang, 2021 [133]	miR-21	A rat PE model.	NR	Up	Regulates inflammation and lung injury.	*PTEN*	No
Shen, 2025 [134]	miR-22-5p	PASMCs and a PE model in SD rats.	NR	Up	Promotes pulmonary thromboinflammatory injury.	*TLR4*	No
Wang, 2018 [135]	miR-27a miR-27b	NR	Plasma samples from patients with acute PE and controls.	Up	NR	NR	Yes (AUC = 0.909)
Zhou, 2016 [136] Mao, 2020 [137]	miR-28-3p	EPCs and PE models in mice and beagle dogs.	Plasma samples from patients with PE and controls.	Up	Regulates apoptosis in EPCs.	*API5*	Yes (AUC = 0.792)
Xiao, 2011 [138]	miR-134	NR	Plasma samples from patients with PE and controls.	Up	NR	NR	Yes (AUC = 0.833)
Zhou, 2021 [139]	miR-190 miR-197	NR	Plasma samples from patients with PE or myocardial infarction and controls.	Up	NR	NR	Yes (AUC = 0.954)
Liu, 2018 [140] Tang, 2024 [141]	miR-221	Human PASMCs.	Plasma samples from patients with PE and controls.	Up	Promotes PASMC proliferation and migration.	*PTEN*	Yes (AUC = 0.823)
Sun, 2019 [142]	miR-514a-5p	A rat PE model.	Plasma samples from patients with PE and controls.	Up	Promotes inflammation, lung injury, and right ventricular hypertrophy.	*CHRDL1*	Yes
Kessler, 2016 [143] Peng, 2020 [144]	miR-1233	NR	Serum samples from patients with PE or non-ST-elevation myocardial infarction and controls, and plasma samples from patients with chronic obstructive pulmonary disease or acute exacerbations, with or without PE.	Up	NR	NR	Yes (AUC = 0.95)

Abbreviations: AUC, area under the receiver operating characteristic curve; CTEPH, chronic thromboembolic pulmonary hypertension; IPAH, idiopathic pulmonary arterial hypertension; PASMC, pulmonary artery smooth muscle cell; PE, pulmonary embolism; NR, not reported. Expression indicates the direction reported in the cited study.

**Table 4:** Antithrombotic miRNAs in pulmonary embolism.

Reference	miRNA	Experimental Model(s)	Human Cohort/Sample	Expression	Principal Reported Effect	Target(s)	Diagnostic Potential
Yang, 2019 [145]	miR-22-3p	HL-1 and AC16 cells and a PE model in SD rats.	NR	Down	Regulates inflammatory responses and pyroptosis.	*MALAT1* *NLRP3*	No
Li, 2022 [146]	miR-34a-3p	Human PASMCs and a rat PE model.	NR	Down	Regulates vascular remodeling.	*DUSP1*	No
Chen, 2020 [147]	miR-106b-5p	Mouse PASMCs and a mouse PE model.	NR	Down	Regulates PASMC proliferation and migration.	*NOR1*	No
Yang, 2025 [148]	miR-124	A rat PE model.	NR	Down	Regulates endothelial metabolic reprogramming.	*PTBP1*	No
Nafady, 2022 [149] Jiang, 2024 [150]	miR-145	PE model in rats.	Plasma samples from patients with chronic obstructive pulmonary disease, with and without PE.	Down	Maintains pulmonary function and reduces myocardial injury.	*IRS1*	Yes
Wu, 2024 [151]	miR-150	HUVECs and plasma samples from patients with DVT and PE.	NR	Down	Promotes cell proliferation, inhibits apoptosis, and reduces inflammation and oxidative stress.	NR	Yes (AUC = 0.912)

Abbreviations: AUC, area under the receiver operating characteristic curve; PASMC, pulmonary artery smooth muscle cell; PE, pulmonary embolism; SD, Sprague-Dawley; NR, not reported. Expression indicates the direction reported in the cited study.

**Table 5:** Prothrombotic miRNAs in venous thromboembolism.

Reference	miRNA	Experimental Model(s)	Human Cohort/Sample	Expression	Principal Reported Effect	Target(s)	Diagnostic Potential
Rossetti, 2021 [154]	miR-126	NR	Plasma samples from patients with VTE and healthy controls.	Up	Activates inflammatory cytokines and inflammatory cells.	NR.	No
Cao, 2018 [155]	miR-130a	Bioinformatic analysis	Bioinformatic analysis	Up	NR	NR	Yes
Wang, 2024 [156]	miR-144	Bioinformatic analysis	Bioinformatic analysis	Up	Promotes inflammatory responses associated with thrombus formation.	*FOS*	No
Fazzalari, 2021 [157]	miR-141-3p miR-200a-3p miR-200c-3p	Plasma samples from hibernating and summer-active black bears.	NR	Down	Inhibit thrombin and other proteases in the coagulation cascade.	*SERPINC1*	Yes
Jian, 2021 [158]	miR-200c-3p	Human embryonic kidney (HEK293) cells and a rat DVT model.	Plasma samples from patients with VTE and controls.	Up	Inhibits antithrombin activity, contributing to thrombus development.	*SERPINC1*	Yes
Erhart, 2024 [159]	miR-451a miR-221-3p	NR	Plasma samples from patients with grade IV glioma, with or without VTE.	Up	Regulates platelets and endothelial cells.	NR	Yes
Zhang, 2023 [160]	miR-483-5p	NR	Plasma samples from patients with lung cancer, with and without VTE.	Up	Activates inflammatory pathways.	NR.	Yes
Li, 2023 [161]	miR-3613-5p	NR	Plasma samples from patients with acute VTE.	Up	Is associated with reduced fibrinogen and coagulation/fibrinolysis complexes and increased platelet aggregation.	NR	Yes (AUC = 0.805)
Gabler, 2021 [162]	miR-6798-3p, miR-6789-5p, miR-4651, miR-6765-5p, miR-6816-5p, miR-4734	NR	Plasma samples from patients at high risk of VTE and healthy controls.	Up	Activation of platelets, coagulation, and endothelial functions.	NR	Yes

Abbreviations: AUC, area under the receiver operating characteristic curve; VTE, venous thromboembolism; NR, not reported. Expression indicates the direction reported in the cited study.

**Table 6:** Antithrombotic miRNAs in venous thromboembolism.

Reference	miRNA	Experimental Model(s)	Human Cohort/Sample	Expression	Principal Reported Effect	Target(s)	Diagnostic Potential
Zeng, 2019 [163]	miR-99a miR-449a	HUVECs and a VTE model in SD rats.	Serum samples from patients with VTE and controls.	Down	Increases thrombomodulin expression and promotes angiogenesis.	*ANRIL*; *BECN1*	Yes
Tian., 2021 [164]	miR-365a-3p	NR	Plasma samples from pregnant women and women using oral contraceptives.	Down	Reduces tissue factor activity and thrombin generation.	*TF*	NR
Lan, 2022 [165]	miR-200a-5p	HUVECs	Plasma samples from patients with VTE and controls.	Down	Enhances endothelial-cell migration and vascular repair.	*COX7C*	Yes
Wang, 2020 [166]	miR-195-5p, miR-205-5p	NR	Plasma samples from patients with cervical cancer treated with chemoradiotherapy alone or with low-molecular-weight heparin.	Up	Regulate angiogenesis.	*VEGF-A*	Yes
Chen, 2021 [167]	miR-522-3p, miR-134	NR	Plasma samples from patients with VTE and controls.	Up	Regulate protein synthesis and apoptosis.	NR	Yes
Morelli, 2024 [168] Teixeira-Costa, 2025 [169]	miR-145	NR	Plasma samples from a population-based cohort and patients with cervical cancer.	Down	Promotes vascular protection and anti-inflammatory effects.	NR	Yes

Abbreviations: HUVEC, human umbilical vein endothelial cell; SD, Sprague-Dawley; TF, tissue factor; VTE, venous thromboembolism; NR, not reported. Expression indicates the direction reported in the cited study.

## Data Availability

All data is available as part of this case report and no additional source data are required.

## Supplementary Materials

The following supporting information can be downloaded at: https://www.mdpi.com/article/10.3390/jcm15166306/s1.

## Abbreviations

VTE	Venous thromboembolism
DVT	Deep vein thrombosis
PE	Pulmonary embolism
PTS	Post-thrombotic syndrome
CTEPH	Chronic thromboembolic pulmonary hypertension
miRNA	MicroRNA
mRNA	Messenger RNA
lncRNA	Long noncoding RNA
ceRNA	Competing endogenous RNA
PRISMA	Preferred Reporting Items for Systematic Reviews and Meta-Analyses
qRT-PCR	Quantitative real-time polymerase chain reaction
TF	Tissue factor
PAI-1	Plasminogen activator inhibitor-1
HUVEC	Human umbilical vein endothelial cell
EPC	Endothelial progenitor cell
PASMC	Pulmonary artery smooth muscle cell
PBMC	Peripheral blood mononuclear cell
AUC	Area under the receiver operating characteristic curve
SNP	Single-nucleotide polymorphism
NF-κB	Nuclear factor kappa B
PI3K/Akt	Phosphoinositide 3-kinase/protein kinase B
MAPK	Mitogen-activated protein kinase
mTOR	Mechanistic target of rapamycin
IPAH	Idiopathic pulmonary arterial hypertension
MM	Multiple myeloma
EC	Endothelial cell
SD	Sprague-Dawley
NR	Not reported

## Nomenclature

Gene symbols are presented in italics in accordance with standard gene nomenclature. For protein-coding genes, the encoded protein is listed below; noncoding genes are identified as producing non-protein-coding RNA. Standardized gene symbols are treated as nomenclature rather than manuscript abbreviations.

**Gene Nomenclature Table**					
**Gene symbol**	**OMIM ID**	**Encoded protein/RNA product**	**Gene symbol**	**OMIM ID**	**Encoded protein/RNA product**
*ABLIM1*	602330	actin-binding LIM protein 1	*MCL1*	159552	*MCL1* apoptosis regulator (*BCL2* family member)
*ANGPT2*	601922	angiopoietin-2	*MDM2*	164785	*MDM2* proto-oncogene protein
*ANRIL*	613149	CDKN2B antisense RNA 1 long noncoding RNA (non-protein-coding RNA product)	*MEG3*	605636	maternally expressed 3 long noncoding RNA (non-protein-coding RNA product)
*API5*	609774	apoptosis inhibitor 5	*MEG8*	613648	maternally expressed 8 long noncoding RNA (non-protein-coding RNA product)
*AR*	313700	androgen receptor	*MMP1*	120353	matrix metallopeptidase 1
*ATG2B*	616226	autophagy related 2B	*MMP2*	120360	matrix metallopeptidase 2
*BAX*	600040	BCL2-associated X protein	*MYB*	189990	*MYB* proto-oncogene, transcription factor
*BCL2*	151430	B-cell lymphoma 2 protein	*MYC*	190080	*MYC* proto-oncogene, bHLH transcription factor
*BCL2L11*	603827	BCL2-like protein 11	*MYD88*	602170	myeloid differentiation primary response protein MyD88
*BDKRB2*	113503	bradykinin receptor B2	*NDUFA4*	603833	*NDUFA4* mitochondrial complex associated
*BECN1*	604378	beclin 1	*NEAT1*	612769	nuclear paraspeckle assembly transcript 1 long noncoding RNA (non-protein-coding RNA product)
*CASC2*	608598	cancer susceptibility 2 long noncoding RNA (non-protein-coding RNA product)	*Nek2*	604043	never in mitosis A-related kinase 2
*CASP1*	147678	caspase-1	*NLRP3*	606416	NLR family pyrin domain containing 3
*CCL2*	158105	C-C motif chemokine ligand 2	*NORAD*	617037	non-coding RNA activated by DNA damage (non-protein-coding RNA product)
*CCL7*	158106	C-C motif chemokine ligand 7	*NOS3*	163729	endothelial nitric oxide synthase
*CD36*	173510	*CD36* molecule (platelet glycoprotein 4)	*NOTCH1*	190198	Notch receptor 1
*Cdca8*	609977	cell division cycle associated 8 (borealin)	*NR4A3*	600542	nuclear receptor subfamily 4 group A member 3 (NOR-1)
*CHRDL1*	300350	chordin-like 1	*NTAN1*	615367	N-terminal asparagine amidase
*COX7C*	603774	cytochrome c oxidase subunit 7C	*P4HB*	176790	protein disulfide-isomerase (prolyl 4-hydroxylase beta subunit)
*CREB1*	123810	cAMP responsive element binding protein 1	*PARP9*	612065	poly(ADP-ribose) polymerase family member 9
*CRNDE*	615624	colorectal neoplasia differentially expressed long noncoding RNA (non-protein-coding RNA product)	*PCYOX1L*	621338	prenylcysteine oxidase 1-like protein
*CXCL12*	600835	C-X-C motif chemokine ligand 12	*Pcyox1l*	621338	prenylcysteine oxidase 1-like protein
*CXCR2*	146928	C-X-C motif chemokine receptor 2	*PDE1B*	171891	phosphodiesterase 1B
*DICER1*	606241	dicer 1, ribonuclease III	*PIK3R2*	603157	phosphoinositide-3-kinase regulatory subunit 2
*DROSHA*	608828	Drosha ribonuclease III	*PRKCA*	176960	protein kinase C alpha
*DUSP1*	600714	dual specificity phosphatase 1	*PRKCD*	176977	protein kinase C delta
*EDNRA*	131243	endothelin receptor type A	*PTBP1*	600693	polypyrimidine tract binding protein 1
*ESM1*	601521	endothelial cell-specific molecule 1	*PTEN*	601728	phosphatase and tensin homolog
*F3*	134390	coagulation factor III (tissue factor)	*PTGS2*	600262	cyclooxygenase-2
*FASLG*	134638	Fas ligand	*PTK2*	600758	focal adhesion kinase 1
*FGF1*	131220	fibroblast growth factor 1	*RAN*	601179	*RAN* GTPase
*FGF5*	165190	fibroblast growth factor 5	*RRAGB*	300725	Ras-related GTP-binding protein B
*FOS*	164810	Fos proto-oncogene, activator protein 1 transcription factor subunit	*RSL24D1*	613262	ribosomal L24 domain containing 1
*FOXO1*	136533	forkhead box O1	*S100A4*	114210	S100 calcium-binding protein A4
*FOXO4*	300033	forkhead box O4	*SERP1*	617674	stress-associated endoplasmic reticulum protein 1
*FOXP3*	300292	forkhead box P3	*SERPINC1*	107300	antithrombin
*GAB2*	606203	GRB2-associated binding protein 2	*SERPINE1*	173360	plasminogen activator inhibitor-1
*GABARAPL1*	607420	gamma-aminobutyric acid type A receptor-associated protein-like 1	*SIRT1*	604479	sirtuin 1
*GAS5*	608280	growth arrest-specific 5 long noncoding RNA (non-protein-coding RNA product)	*SNHG12*	621451	small nucleolar RNA host gene 12 long noncoding RNA (non-protein-coding RNA product)
*GJA1*	121014	gap junction protein alpha 1 (connexin 43)	*SPRED1*	609291	sprouty-related EVH1 domain-containing protein 1
*HES1*	139605	hes family bHLH transcription factor 1	*SRCIN1*	610786	SRC kinase signaling inhibitor 1
*HEY2*	604674	hes-related family bHLH transcription factor with YRPW motif 2	*SRF*	600589	serum response factor
*HIF1A*	603348	hypoxia-inducible factor 1 alpha subunit	*STAT3*	102582	signal transducer and activator of transcription 3
*HMGB1*	163905	high mobility group box 1	*TAB2*	605101	TGF-beta activated kinase 1 binding protein 2
*Hmgcr*	142910	3-hydroxy-3-methylglutaryl-CoA reductase	*TGFB1*	190180	transforming growth factor beta 1
*HSF2*	140581	heat shock transcription factor 2	*TGFB2*	190220	transforming growth factor beta 2
*IGF1*	147440	insulin-like growth factor 1	*TIMD4*	610096	T-cell immunoglobulin and mucin domain-containing protein 4
*IL10*	124092	interleukin-10	*TLR2*	603028	Toll-like receptor 2
*IL1R1*	147810	interleukin-1 receptor type 1	*TLR4*	603030	Toll-like receptor 4
*IL6*	147620	interleukin-6	*TLR7*	300365	Toll-like receptor 7
*IL6R*	147880	interleukin-6 receptor	*TNFSF13B*	603969	tumor necrosis factor ligand superfamily member 13B
*IRS1*	147545	insulin receptor substrate 1	*TP53*	191170	tumor protein p53
*JAG1*	601920	Jagged 1	*TRPM7*	605692	transient receptor potential cation channel subfamily M member 7
*JAK2*	147796	Janus kinase 2	*TUG1*	614971	taurine upregulated gene 1 long noncoding RNA (non-protein-coding RNA product)
*JUN*	165160	Jun proto-oncogene, activator protein 1 transcription factor subunit	*TXNIP*	606599	thioredoxin-interacting protein
*Kif18a*	611271	kinesin family member 18A	*UXT-AS1*	N/A	UXT antisense RNA 1 (non-protein-coding RNA product)
*LINC00659*	N/A	long intergenic non-protein coding RNA 659 (non-protein-coding RNA product)	*VAMP8*	603177	vesicle-associated membrane protein 8
*LINC01123*	N/A	long intergenic non-protein coding RNA 1123 (non-protein-coding RNA product)	*VEGFA*	192240	vascular endothelial growth factor A
*LITAF*	603795	lipopolysaccharide-induced tumor necrosis factor-alpha factor	*WNT3A*	606359	Wnt family member 3A
*MALAT1*	607924	metastasis associated lung adenocarcinoma transcript 1 long noncoding RNA (non-protein-coding RNA product)	*WTAPP1*	N/A	WTAP pseudogene 1 (non-protein-coding transcript; no encoded protein)
*MAPK1*	176948	mitogen-activated protein kinase 1	*XPO5*	607845	exportin 5
