# Supplementary material for: The Role of Circulating MicroRNAs as Biomarkers and Therapeutic Targets in Venous Thromboembolism: A Systematic Review
Source: J Clin Med. 2026 Aug 14;15(16):6306. doi: 10.3390/jcm15166306 (PMC13513252; doi:10.3390/jcm15166306)
Supplement: Supplementary file 1 [file jcm-15-06306-s001.zip › Supplementary Table S2.pdf]

Supplementary Table S2. Risk-of-bias assessment of included in vitro studies using the QUIN tool.

Study	C1	C2	C3	C4	C5	C6	C7	C8	C9	C10	C11	C12	Total	QUIN score (%)	Risk of bias
Deep vein thrombosis (DVT) studies															
Feng et al., 2022 [32]	2	0	1	2	2	0	0	2	0	0	2	2	**13**	**54.2%**	**Moderate**
Zhou et al., 2024 [33]	2	0	1	2	1	0	0	2	0	0	1	2	**11**	**45.8%**	**High**
Wang et al., 2025 [34]	2	0	1	2	2	0	0	2	0	0	2	2	**13**	**54.2%**	**Moderate**
Xie et al., 2016 [35]	2	0	2	2	2	0	0	2	0	0	1	1	**12**	**50.0%**	**Moderate**
Xu et al., 2022 [36]	2	0	2	2	2	0	1	2	0	0	1	2	**14**	**58.3%**	**Moderate**
Huang et al., 2023 [37]	2	0	1	2	2	0	0	2	0	0	1	2	**12**	**50.0%**	**Moderate**
Yu et al., 2023 [38]	2	0	1	2	2	0	0	2	0	0	1	2	**12**	**50.0%**	**Moderate**
Han et al., 2023 [39]	2	0	1	2	1	0	0	2	0	0	1	1	**10**	**41.7%**	**High**
Zhang et al., 2018 [40]	2	0	1	2	2	0	0	2	0	0	1	2	**12**	**50.0%**	**Moderate**
Wang et al., 2025 [41]	2	0	1	2	1	0	0	2	0	0	1	2	**11**	**45.8%**	**High**
Qin et al., 2015 [42]	2	0	2	2	2	0	0	2	0	0	1	2	**13**	**54.2%**	**Moderate**
Mo et al., 2016 [43]	2	0	2	2	2	0	0	2	0	0	1	1	**12**	**50.0%**	**Moderate**
Qin et al., 2018 [44]	2	0	1	2	1	0	0	2	0	0	1	1	**10**	**41.7%**	**High**
Jin et al., 2019 [45]	2	0	1	1	2	0	0	2	0	0	1	2	**11**	**45.8%**	**High**
Tian et al., 2022 [46]	2	0	1	2	1	0	0	2	0	0	1	1	**10**	**41.7%**	**High**
Zhou et al., 2024 [47]	2	0	1	2	1	0	0	2	0	0	1	2	**11**	**45.8%**	**High**
Li et al., 2022 [48]	2	0	2	2	2	0	0	2	0	0	1	1	**12**	**50.0%**	**Moderate**
Jiang et al., 2017 [49]	2	0	2	2	2	0	0	2	0	0	1	1	**12**	**50.0%**	**Moderate**
Srivastava et al., 2019 [50]	2	0	1	2	2	0	0	2	0	0	1	1	**11**	**45.8%**	**High**
Srivastava et al., 2024 [51]	2	0	1	2	2	0	1	2	0	0	0	1	**11**	**45.8%**	**High**
Tang et al., 2021 [52]	2	0	1	2	1	0	0	2	0	0	1	1	**10**	**41.7%**	**High**
Zhang et al., 2020 [53]	2	0	1	2	2	0	0	2	0	0	1	2	**12**	**50.0%**	**Moderate**
Wang et al., 2022 [54]	2	0	1	2	1	0	0	2	0	0	1	1	**10**	**41.7%**	**High**
Wang et al., 2016 [55]	2	0	2	2	2	0	0	2	0	0	1	2	**13**	**54.2%**	**Moderate**
Xiao et al., 2024 [56]	2	0	1	2	2	0	0	2	0	0	1	2	**12**	**50.0%**	**Moderate**
Wang et al., 2022 [57]	2	0	1	1	1	0	0	2	0	0	1	1	**9**	**37.5%**	**High**
Wang et al., 2021 [58]	2	0	1	1	2	0	0	2	0	0	1	1	**10**	**41.7%**	**High**
Guo et al., 2025 [59]	2	0	1	2	2	0	0	2	0	0	1	2	**12**	**50.0%**	**Moderate**
Lu et al., 2019 [60]	2	0	1	2	2	0	0	2	0	0	1	1	**11**	**45.8%**	**High**
Zhang et al., 2024 [61]	2	0	1	2	1	0	0	2	0	0	1	2	**11**	**45.8%**	**High**
Kong et al., 2016 [62]	2	0	2	2	2	0	0	2	0	0	1	1	**12**	**50.0%**	**Moderate**
Zhu et al., 2020 [63]	2	0	1	2	1	0	0	2	0	0	1	1	**10**	**41.7%**	**High**
Zhou et al., 2020 [64]	2	0	1	2	1	0	0	2	0	0	1	1	**10**	**41.7%**	**High**
Wang et al., 2018 [65]	2	0	1	2	1	0	0	2	0	0	1	1	**10**	**41.7%**	**High**
Du et al., 2019 [66]	2	0	1	2	2	0	0	2	0	0	1	1	**11**	**45.8%**	**High**
Qiao et al., 2024 [67]	2	0	1	2	1	0	0	2	0	0	1	1	**10**	**41.7%**	**High**
Wang et al., 2026 [68]	2	0	1	2	2	0	1	2	0	0	1	1	**12**	**50.0%**	**Moderate**
Liang et al., 2020 [69]	2	0	1	2	1	0	0	2	0	0	1	1	**10**	**41.7%**	**High**
Lou et al., 2022 [70]	2	0	1	2	1	0	0	1	0	0	1	1	**9**	**37.5%**	**High**
Li et al., 2017 [71]	2	0	1	2	1	0	0	2	0	0	1	1	**10**	**41.7%**	**High**
Rahmanpour et al., 2022 [72]	2	0	1	2	1	0	0	1	0	0	1	1	**9**	**37.5%**	**High**
Lou et al., 2022 [73]	2	0	1	2	2	0	0	2	0	0	1	1	**11**	**45.8%**	**High**

Study	C1	C2	C3	C4	C5	C6	C7	C8	C9	C10	C11	C12	Total	QUIN score (%)	Risk of bias
Sun et al., 2020 [74]	2	0	1	2	1	0	0	2	0	0	1	1	**10**	**41.7%**	**High**
Cao et al., 2022 [75]	2	0	1	2	1	0	0	2	0	0	1	1	**10**	**41.7%**	**High**
Zhang et al., 2020 [76]	2	0	1	2	1	0	0	2	0	0	1	1	**10**	**41.7%**	**High**
Li et al., 2020 [77]	2	0	1	2	1	0	0	2	0	0	1	1	**10**	**41.7%**	**High**
Yang et al., 2022 [78]	2	0	1	2	2	0	0	2	0	0	1	1	**11**	**45.8%**	**High**
Meng et al., 2015 [79]	2	0	1	2	2	0	0	2	0	0	1	2	**12**	**50.0%**	**Moderate**
Chen et al., 2016 [80]	2	0	1	1	2	0	0	2	0	0	1	1	**10**	**41.7%**	**High**
Zhu et al., 2023 [81]	2	0	1	2	2	0	0	2	0	0	1	2	**12**	**50.0%**	**Moderate**
Ou et al., 2020 [82]	2	0	1	2	2	0	0	2	0	0	1	2	**12**	**50.0%**	**Moderate**
Feng et al., 2022 [83]	2	0	1	2	2	0	0	2	0	0	1	1	**11**	**45.8%**	**High**
Chen et al., 2025 [84]	2	0	1	2	1	0	1	1	0	0	1	1	**10**	**41.7%**	**High**
Zhang et al., 2020 [85]	2	0	1	2	2	0	0	2	0	0	1	2	**12**	**50.0%**	**Moderate**
Jha et al., 2021 [86]	2	0	1	2	1	0	0	2	0	0	1	1	**10**	**41.7%**	**High**
Sahu et al., 2017 [87]	2	0	1	2	2	0	0	2	0	0	1	2	**12**	**50.0%**	**Moderate**
Antoun et al., 2024 [88]	2	2	2	2	2	1	0	2	2	2	2	2	**21**	**87.5%**	**Low**
Wang et al., 2024 [89]	2	0	1	2	2	0	0	2	0	0	1	2	**12**	**50.0%**	**Moderate**
Wang et al., 2014 [90]	2	0	1	2	2	0	0	2	0	0	1	2	**12**	**50.0%**	**Moderate**
Wang et al., 2019 [91]	2	0	1	2	2	0	0	2	0	0	1	2	**12**	**50.0%**	**Moderate**
Du et al., 2020 [92]	2	0	1	2	2	0	0	2	0	0	1	2	**12**	**50.0%**	**Moderate**
He et al., 2023 [93]	2	0	1	2	2	0	0	2	0	0	2	2	**13**	**54.2%**	**Moderate**
Zhang et al., 2021 [94]	*NA*	*NA*	*NA*	*NA*	*NA*	*NA*	*NA*	*NA*	*NA*	*NA*	*NA*	*NA*	***NA***	***NA***	***Not assessed***
Zhang et al., 2024 [95]	2	0	0	2	1	0	0	2	0	0	1	1	**9**	**37.5%**	**High**
Yang et al., 2023 [96]	2	0	2	2	2	1	0	2	0	0	1	1	**13**	**54.2%**	**Moderate**
Lu et al., 2022 [97]	2	0	1	1	1	0	0	2	0	0	2	1	**10**	**41.7%**	**High**
Ni et al., 2023 [98]	2	0	1	2	1	1	0	2	0	0	1	1	**11**	**45.8%**	**High**
Ding et al., 2022 [99]	2	0	2	2	2	1	0	2	0	0	1	1	**13**	**54.2%**	**Moderate**
Chen et al., 2023 [100]	2	0	2	2	2	1	0	2	0	0	2	1	**14**	**58.3%**	**Moderate**
Sun et al., 2019 [101]	2	0	0	2	2	1	0	2	0	0	1	1	**11**	**45.8%**	**High**
Wang et al., 2023 [102]	2	0	2	2	2	0	0	2	0	0	2	1	**13**	**54.2%**	**Moderate**
Li et al., 2020 [103]	*NA*	*NA*	*NA*	*NA*	*NA*	*NA*	*NA*	*NA*	*NA*	*NA*	*NA*	*NA*	***NA***	***NA***	***Not assessed***
Sun et al., 2020 [104]	2	0	2	2	2	0	0	2	0	0	1	2	**13**	**54.2%**	**Moderate**
Xu et al., 2022 [105]	*NA*	*NA*	*NA*	*NA*	*NA*	*NA*	*NA*	*NA*	*NA*	*NA*	*NA*	*NA*	***NA***	***NA***	***Not assessed***
Luo et al., 2025 [106]	2	0	2	2	1	0	0	2	0	0	2	2	**13**	**54.2%**	**Moderate**
Pan et al., 2021 [107]	2	0	2	2	1	1	0	2	0	0	1	1	**12**	**50.0%**	**Moderate**
Zhang et al., 2025 [108]	2	0	2	2	1	1	0	2	0	0	1	1	**12**	**50.0%**	**Moderate**
Zhang et al., 2020 [109]	2	0	2	2	2	0	0	2	0	0	2	2	**14**	**58.3%**	**Moderate**
Pan et al., 2022 [110]	2	0	2	2	2	0	0	2	0	0	2	1	**13**	**54.2%**	**Moderate**
Yang et al., 2020 [111]	2	0	2	2	2	1	0	2	0	0	1	2	**14**	**58.3%**	**Moderate**
Ai et al., 2018 [112]	2	0	2	2	2	0	0	2	0	0	1	1	**12**	**50.0%**	**Moderate**
Marchand et al., 2012 [113]	2	0	1	2	2	1	0	2	1	0	1	2	**14**	**58.3%**	**Moderate**
Chang et al., 2019 [114]	*NA*	*NA*	*NA*	*NA*	*NA*	*NA*	*NA*	*NA*	*NA*	*NA*	*NA*	*NA*	***NA***	***NA***	***Not assessed***
Kong et al., 2016 [115]	2	0	1	2	2	1	0	2	0	0	1	2	**13**	**54.2%**	**Moderate**
Fan et al., 2024 [116]	2	0	1	2	2	1	0	2	0	0	1	2	**13**	**54.2%**	**Moderate**
Tang et al., 2018 [117]	2	0	1	2	2	1	0	2	0	0	1	2	**13**	**54.2%**	**Moderate**

Study	C1	C2	C3	C4	C5	C6	C7	C8	C9	C10	C11	C12	Total	QUIN score (%)	Risk of bias
Xu et al., 2025 [118]	2	0	1	2	2	1	0	2	0	0	1	2	**13**	**54.2%**	**Moderate**
Chu et al., 2022 [119]	2	0	1	2	2	1	0	2	0	0	2	2	**14**	**58.3%**	**Moderate**
Zhu et al., 2023 [120]	2	0	1	2	2	1	0	2	0	0	1	2	**13**	**54.2%**	**Moderate**
Wang et al., 2018 [121]	*NA*	*NA*	*NA*	*NA*	*NA*	*NA*	*NA*	*NA*	*NA*	*NA*	*NA*	*NA*	***NA***	***NA***	***Not assessed***
Qui et al., 2024 [122]	2	0	1	2	2	1	0	2	0	0	1	2	**13**	**54.2%**	**Moderate**
Li et al., 2018 [123]	2	0	1	2	2	1	0	2	0	0	1	2	**13**	**54.2%**	**Moderate**
Lu et al., 2021 [124]	2	0	1	2	2	1	0	2	0	0	1	2	**13**	**54.2%**	**Moderate**
Wang et al., 2023 [125]	*NA*	*NA*	*NA*	*NA*	*NA*	*NA*	*NA*	*NA*	*NA*	*NA*	*NA*	*NA*	***NA***	***NA***	***Not assessed***
Jin et al., 2017 [126]	*NA*	*NA*	*NA*	*NA*	*NA*	*NA*	*NA*	*NA*	*NA*	*NA*	*NA*	*NA*	***NA***	***NA***	***Not assessed***
Hao et al., 2024 [127]	*NA*	*NA*	*NA*	*NA*	*NA*	*NA*	*NA*	*NA*	*NA*	*NA*	*NA*	*NA*	***NA***	***NA***	***Not assessed***
Zhang et al., 2025 [128]	*NA*	*NA*	*NA*	*NA*	*NA*	*NA*	*NA*	*NA*	*NA*	*NA*	*NA*	*NA*	***NA***	***NA***	***Not assessed***
Pan et al., 2025 [129]	*NA*	*NA*	*NA*	*NA*	*NA*	*NA*	*NA*	*NA*	*NA*	*NA*	*NA*	*NA*	***NA***	***NA***	***Not assessed***
Shi et al., 2025 [130]	*NA*	*NA*	*NA*	*NA*	*NA*	*NA*	*NA*	*NA*	*NA*	*NA*	*NA*	*NA*	***NA***	***NA***	***Not assessed***
Pulmonary embolism (PE) studies															
Liu et al., 2020 [131]	2	0	1	2	2	0	0	2	0	0	1	2	**12**	**50.0%**	**Moderate**
Zhang et al., 2022 [132]	2	0	1	2	2	1	0	2	1	0	2	2	**15**	**62.5%**	**Moderate**
Fabro et al., 2021 [133]	*NA*	*NA*	*NA*	*NA*	*NA*	*NA*	*NA*	*NA*	*NA*	*NA*	*NA*	*NA*	***NA***	***NA***	***Not assessed***
Liang et al., 2021 [134]	*NA*	*NA*	*NA*	*NA*	*NA*	*NA*	*NA*	*NA*	*NA*	*NA*	*NA*	*NA*	***NA***	***NA***	***Not assessed***
Shen et al., 2025 [135]	2	1	1	2	2	1	1	2	0	0	2	1	**15**	**62.5%**	**Moderate**
Wang et al., 2018 [136]	*NA*	*NA*	*NA*	*NA*	*NA*	*NA*	*NA*	*NA*	*NA*	*NA*	*NA*	*NA*	***NA***	***NA***	***Not assessed***
Zhou et al., 2016 [137]	*NA*	*NA*	*NA*	*NA*	*NA*	*NA*	*NA*	*NA*	*NA*	*NA*	*NA*	*NA*	***NA***	***NA***	***Not assessed***
Mao et al., 2020 [138]	2	0	1	1	2	0	1	2	0	0	1	1	**11**	**45.8%**	**High**
Xiao et al., 2011 [139]	*NA*	*NA*	*NA*	*NA*	*NA*	*NA*	*NA*	*NA*	*NA*	*NA*	*NA*	*NA*	***NA***	***NA***	***Not assessed***
Zhou et al., 2021 [140]	*NA*	*NA*	*NA*	*NA*	*NA*	*NA*	*NA*	*NA*	*NA*	*NA*	*NA*	*NA*	***NA***	***NA***	***Not assessed***
Liu et al., 2018 [141]	*NA*	*NA*	*NA*	*NA*	*NA*	*NA*	*NA*	*NA*	*NA*	*NA*	*NA*	*NA*	***NA***	***NA***	***Not assessed***
Tang et al., 2024 [142]	2	0	1	2	2	1	0	2	1	1	1	2	**15**	**62.5%**	**Moderate**
Sun et al., 2019 [143]	*NA*	*NA*	*NA*	*NA*	*NA*	*NA*	*NA*	*NA*	*NA*	*NA*	*NA*	*NA*	***NA***	***NA***	***Not assessed***
Kessler et al., 2016 [144]	*NA*	*NA*	*NA*	*NA*	*NA*	*NA*	*NA*	*NA*	*NA*	*NA*	*NA*	*NA*	***NA***	***NA***	***Not assessed***
Peng et al., 2020 [145]	*NA*	*NA*	*NA*	*NA*	*NA*	*NA*	*NA*	*NA*	*NA*	*NA*	*NA*	*NA*	***NA***	***NA***	***Not assessed***
Yang et al., 2019 [146]	2	0	1	2	1	1	0	2	0	0	1	1	**11**	**45.8%**	**High**
Li et al., 2022 [147]	2	0	1	2	2	1	0	2	0	0	1	2	**13**	**54.2%**	**Moderate**
Song et al., 2024 [148]	2	0	1	2	2	1	0	2	0	0	1	2	**13**	**54.2%**	**Moderate**
Chen et al., 2020 [149]	2	0	1	2	2	1	0	2	0	0	2	1	**13**	**54.2%**	**Moderate**
Yang et al., 2025 [150]	*NA*	*NA*	*NA*	*NA*	*NA*	*NA*	*NA*	*NA*	*NA*	*NA*	*NA*	*NA*	***NA***	***NA***	***Not assessed***
Nafady et al., 2022 [151]	*NA*	*NA*	*NA*	*NA*	*NA*	*NA*	*NA*	*NA*	*NA*	*NA*	*NA*	*NA*	***NA***	***NA***	***Not assessed***
Jiang et al., 2024 [152]	*NA*	*NA*	*NA*	*NA*	*NA*	*NA*	*NA*	*NA*	*NA*	*NA*	*NA*	*NA*	***NA***	***NA***	***Not assessed***
Wu et al., 2024 [153]	2	0	1	2	2	0	0	2	0	0	1	1	**11**	**45.8%**	**High**
Oto et al., 2020 [154]	*NA*	*NA*	*NA*	*NA*	*NA*	*NA*	*NA*	*NA*	*NA*	*NA*	*NA*	*NA*	***NA***	***NA***	***Not assessed***
Zhu et al., 2022 [155]	2	0	1	2	2	0	0	2	0	0	1	1	**11**	**45.8%**	**High**
Venous thromboembolism (VTE) studies															
Rossetti et al., 2021 [156]	*NA*	*NA*	*NA*	*NA*	*NA*	*NA*	*NA*	*NA*	*NA*	*NA*	*NA*	*NA*	***NA***	***NA***	***Not assessed***
Cao et al., 2018 [157]	*NA*	*NA*	*NA*	*NA*	*NA*	*NA*	*NA*	*NA*	*NA*	*NA*	*NA*	*NA*	***NA***	***NA***	***Not assessed***
Wang et al., 2024 [158]	2	0	1	2	2	0	0	2	0	0	1	1	**11**	**45.8%**	**High**
Fazzalari et al., 2021 [159]	2	0	1	2	2	0	0	2	0	0	0	1	**10**	**41.7%**	**High**

Study	C1	C2	C3	C4	C5	C6	C7	C8	C9	C10	C11	C12	Total	QUIN score (%)	Risk of bias
Jian et al., 2021 [160]	2	0	1	2	2	0	0	2	0	0	2	1	**12**	**50.0%**	**Moderate**
Erhart et al., 2024 [161]	*NA*	*NA*	*NA*	*NA*	*NA*	*NA*	*NA*	*NA*	*NA*	*NA*	*NA*	*NA*	***NA***	***NA***	***Not assessed***
Zhang et al., 2023 [162]	*NA*	*NA*	*NA*	*NA*	*NA*	*NA*	*NA*	*NA*	*NA*	*NA*	*NA*	*NA*	***NA***	***NA***	***Not assessed***
Li et al., 2023 [163]	2	0	1	1	2	0	0	2	0	0	1	1	**10**	**41.7%**	**High**
Gabler et al., 2021 [164]	*NA*	*NA*	*NA*	*NA*	*NA*	*NA*	*NA*	*NA*	*NA*	*NA*	*NA*	*NA*	***NA***	***NA***	***Not assessed***
Zeng et al., 2019 [165]	2	0	1	2	2	0	0	2	0	0	1	1	**11**	**45.8%**	**High**
Tian et al., 2021 [166]	2	0	1	2	2	0	0	2	0	0	1	2	**12**	**50.0%**	**Moderate**
Lan et al., 2022 [167]	2	0	1	2	2	0	0	2	0	0	0	1	**10**	**41.7%**	**High**
Wang et al., 2020 [168]	*NA*	*NA*	*NA*	*NA*	*NA*	*NA*	*NA*	*NA*	*NA*	*NA*	*NA*	*NA*	***NA***	***NA***	***Not assessed***
Chen et al., 2021 [169]	*NA*	*NA*	*NA*	*NA*	*NA*	*NA*	*NA*	*NA*	*NA*	*NA*	*NA*	*NA*	***NA***	***NA***	***Not assessed***
Morelli et al., 2024 [170]	*NA*	*NA*	*NA*	*NA*	*NA*	*NA*	*NA*	*NA*	*NA*	*NA*	*NA*	*NA*	***NA***	***NA***	***Not assessed***
Teixeira-Costa et al., 2025 [171]	*NA*	*NA*	*NA*	*NA*	*NA*	*NA*	*NA*	*NA*	*NA*	*NA*	*NA*	*NA*	***NA***	***NA***	***Not assessed***
Ciccacci et al., 2015 [172]	*NA*	*NA*	*NA*	*NA*	*NA*	*NA*	*NA*	*NA*	*NA*	*NA*	*NA*	*NA*	***NA***	***NA***	***Not assessed***
Starikova et al., 2015 [173]	*NA*	*NA*	*NA*	*NA*	*NA*	*NA*	*NA*	*NA*	*NA*	*NA*	*NA*	*NA*	***NA***	***NA***	***Not assessed***
Wang et al., 2019 [174]	*NA*	*NA*	*NA*	*NA*	*NA*	*NA*	*NA*	*NA*	*NA*	*NA*	*NA*	*NA*	***NA***	***NA***	***Not assessed***
Kim et al., 2019 [175]	*NA*	*NA*	*NA*	*NA*	*NA*	*NA*	*NA*	*NA*	*NA*	*NA*	*NA*	*NA*	***NA***	***NA***	***Not assessed***
Ko et al., 2019 [176]	*NA*	*NA*	*NA*	*NA*	*NA*	*NA*	*NA*	*NA*	*NA*	*NA*	*NA*	*NA*	***NA***	***NA***	***Not assessed***
Thibord et al., 2020 [177]	*NA*	*NA*	*NA*	*NA*	*NA*	*NA*	*NA*	*NA*	*NA*	*NA*	*NA*	*NA*	***NA***	***NA***	***Not assessed***
Rodriguez-Rius et al., 2020 [178]	*NA*	*NA*	*NA*	*NA*	*NA*	*NA*	*NA*	*NA*	*NA*	*NA*	*NA*	*NA*	***NA***	***NA***	***Not assessed***
Oto et al., 2020 [179]	*NA*	*NA*	*NA*	*NA*	*NA*	*NA*	*NA*	*NA*	*NA*	*NA*	*NA*	*NA*	***NA***	***NA***	***Not assessed***
Anjis et al., 2022 [180]	*NA*	*NA*	*NA*	*NA*	*NA*	*NA*	*NA*	*NA*	*NA*	*NA*	*NA*	*NA*	***NA***	***NA***	***Not assessed***
Fang et al., 2022 [181]	*NA*	*NA*	*NA*	*NA*	*NA*	*NA*	*NA*	*NA*	*NA*	*NA*	*NA*	*NA*	***NA***	***NA***	***Not assessed***
Ten Cate et al., 2023 [182]	*NA*	*NA*	*NA*	*NA*	*NA*	*NA*	*NA*	*NA*	*NA*	*NA*	*NA*	*NA*	***NA***	***NA***	***Not assessed***
Su et al., 2021 [183]	*NA*	*NA*	*NA*	*NA*	*NA*	*NA*	*NA*	*NA*	*NA*	*NA*	*NA*	*NA*	***NA***	***NA***	***Not assessed***
Rouka et al., 2023 [184]	*NA*	*NA*	*NA*	*NA*	*NA*	*NA*	*NA*	*NA*	*NA*	*NA*	*NA*	*NA*	***NA***	***NA***	***Not assessed***
Chancal et al., 2024 [185]	*NA*	*NA*	*NA*	*NA*	*NA*	*NA*	*NA*	*NA*	*NA*	*NA*	*NA*	*NA*	***NA***	***NA***	***Not assessed***
Zhang et al., 2024 [186]	*NA*	*NA*	*NA*	*NA*	*NA*	*NA*	*NA*	*NA*	*NA*	*NA*	*NA*	*NA*	***NA***	***NA***	***Not assessed***
Lin et al., 2025 [187]	*NA*	*NA*	*NA*	*NA*	*NA*	*NA*	*NA*	*NA*	*NA*	*NA*	*NA*	*NA*	***NA***	***NA***	***Not assessed***

Notes: C1, clearly stated aims and objectives; C2, detailed explanation of sample-size calculation; C3, detailed explanation of sampling technique; C4, details of the comparison group; C5, details of methodology; C6, operator details; C7, randomization; C8, outcome-measurement details; C9, outcome-assessor details; C10, blinding; C11, statistical analysis; C12, presentation of results. Scoring: 2 = adequately specified; 1 = inadequately specified; 0 = not specified; NA = not applicable or not assessed. The QUIN score was calculated as (total score × 100)/(2 × number of applicable criteria). Risk-of-bias categories were low (>70%), moderate (50–70%), and high (<50%).

Abbreviations: DVT, deep vein thrombosis; NA, not applicable; PE, pulmonary embolism; QUIN, Quality Assessment Tool for In Vitro Studies; VTE, venous thromboembolism.
