# Supplementary material for: The Role of Circulating MicroRNAs as Biomarkers and Therapeutic Targets in Venous Thromboembolism: A Systematic Review
Source: J Clin Med. 2026 Aug 14;15(16):6306. doi: 10.3390/jcm15166306 (PMC13513252; doi:10.3390/jcm15166306)
Supplement: Supplementary file 1 [file jcm-15-06306-s001.zip › Supplementary Table S3.pdf]

Supplementary Table S3. Risk-of-bias assessment of animal studies using the SYRCLE tool.

L = low risk; **U** = unclear risk; **H** = high risk; **N/A** = not applicable; **—** = no rating entered in the source table.

Study	SYRCLE risk-of-bias domains									
	Sequence generation	Baseline characteristics	Allocation concealment	Random housing	Caregiver/ investigator blinding	Random outcome assessment	Outcome-assessor blinding	Incomplete outcome data	Selective reporting	Other bias
Feng, 2022 [32]	U	U	U	U	U	U	U	L	U	H
Wang, 2025 [34]	U	L	U	U	U	U	U	U	U	H
Yu, 2023 [38]	U	L	U	U	U	U	U	L	U	H
Zhang, 2018 [40]	U	L	U	U	U	U	U	L	U	H
Li, 2022 [48]	U	L	U	U	U	U	U	L	U	U
Srivastava, 2024 [51]	U	L	U	U	U	U	U	L	U	H
Zhang, 2020 [53]	U	L	U	U	U	U	U	H	U	H
Wang, 2021 [58]	U	L	U	U	U	U	U	U	U	H
Lu, 2019 [60]	U	L	U	U	U	U	U	L	U	H
Zhang, 2024 [61]	U	L	U	U	U	U	U	H	U	H
Kong, 2016 [62]	U	L	U	U	U	U	U	L	U	U
Zhou, 2020 [64]	U	U	U	U	U	U	U	U	U	H
Du, 2019 [66]	U	U	U	U	U	U	U	U	U	H
Qiao, 2024 [67]	H	U	U	U	H	U	H	H	U	H
Wang, 2026 [68]	U	U	U	U	U	U	U	H	U	H
Lou, 2022 [73]	U	U	U	U	U	U	U	U	U	H
Sun, 2020 [74]	U	U	U	U	U	U	U	H	U	H
Yang, 2022 [78]	U	U	U	U	U	U	U	H	H	H
Meng, 2015 [79]	H	U	U	U	U	U	H	H	U	H
Chen, 2016 [80]	U	U	U	U	U	U	U	H	U	H
Ou, 2020 [82]	U	U	U	U	U	U	U	H	U	H
Feng, 2022 [83]	U	U	U	U	U	U	U	U	U	H
Chen, 2025 [84]	U	U	U	U	U	U	U	U	H	H
Sahu, 2017 [87]	U	U	U	U	U	U	U	U	H	H
Wang, 2024 [89]	U	U	U	U	U	U	U	L	H	H
Wang, 2014 [90]	U	U	U	U	U	U	U	L	L	H
Wang, 2019 [91]	U	U	U	U	U	U	U	U	U	H

Study	SYRCLE risk-of-bias domains									
	Sequence generation	Baseline characteristics	Allocation concealment	Random housing	Caregiver/ investigator blinding	Random outcome assessment	Outcome-assessor blinding	Incomplete outcome data	Selective reporting	Other bias
Du, 2020 [92]	U	U	U	U	U	U	U	U	L	H
He, 2023 [93]	U	U	U	U	U	U	U	L	L	H
Lu et al., 2022 [97]	U	U	U	U	U	U	U	H	H	H
Ni et al., 2023 [98]	U	U	U	U	U	U	U	U	U	H
Ding et al., 2022 [99]	U	U	U	U	U	U	U	L	L	H
Sun et al., 2019 [101]	U	U	U	U	U	U	U	U	L	H
Li et al., 2020 [103]	U	U	U	U	U	U	U	U	H	H
Sun et al., 2020 [104]	U	U	U	U	U	U	U	U	L	H
Luo et al., 2025 [106]	U	U	U	U	U	U	U	H	U	H
Pan et al., 2021 [107]	U	U	U	U	U	U	U	L	U	H
Zhang et al., 2020 [109]	U	U	U	U	U	U	U	H	U	U
Pan et al., 2022 [110]	U	U	U	U	U	U	U	L	U	U
Yang et al., 2020 [111]	U	U	U	U	U	U	U	L	U	H
Ai et al., 2018 [112]	U	U	U	U	U	U	U	U	U	H
Kong et al., 2016 [115]	U	U	U	U	U	U	U	L	U	U
Fan et al., 2024 [116]	U	U	U	U	U	U	U	L	U	H
Tang et al., 2018 [117]	U	U	U	U	U	U	U	H	U	H
Xu et al., 2025 [118]	U	U	U	U	U	U	U	H	U	H
Chu et al., 2022 [119]	U	U	U	U	U	U	U	H	U	H
Qui et al., 2024 [122]	U	U	U	U	U	U	U	U	U	H
Li et al., 2018 [123]	U	U	U	U	U	U	U	L	U	U
Lu et al., 2021 [124]	U	U	U	U	U	U	U	L	U	H
Jin et al., 2017 [126]	U	U	U	U	U	U	U	U	U	H
Hao et al., 2024 [127]	U	U	U	U	U	U	U	H	U	H
Liu, 2020 [131]	U	U	U	U	H	U	U	H	U	H
Liang, 2021 [134]	U	L	U	U	H	U	U	H	U	H
Shen, 2025 [135]	U	U	U	U	H	U	U	U	U	H
Zhou, 2016 [137]	U	U	U	U	H	U	U	L	U	H
Mao, 2020 [138]	U	L	U	U	H	U	U	U	H	H

Study	SYRCLE risk-of-bias domains									
	Sequence generation	Baseline characteristics	Allocation concealment	Random housing	Caregiver/ investigator blinding	Random outcome assessment	Outcome-assessor blinding	Incomplete outcome data	Selective reporting	Other bias
Sun, 2019 [143]	U	U	U	U	H	U	H	U	U	H
Yang, 2019 [146]	U	U	U	U	H	U	H	U	H	H
Li, 2022 [147]	U	U	U	U	H	U	H	L	U	H
Song, 2024 [148]	U	U	U	U	H	U	H	H	U	H
Chen, 2020 [149]	U	U	U	U	H	U	H	U	H	H
Yang, 2025 [150]	U	L	U	U	H	U	H	U	U	H
Jiang, 2024 [152]	U	U	U	U	U	U	U	U	U	H
Zhu et al., 2022 [155]	U	U	U	U	U	U	U	U	H	H
Fazzalari, 2021 [159]	U	H	U	U	U	U	U	L	U	H
Jian, 2021 [160]	U	U	U	U	U	U	U	U	U	H
Zeng et al., 2019 [165]	U	U	U	U	U	U	U	U	H	H

Overall domain-level distribution

Summary	Sequence generation	Baseline characteristics	Allocation concealment	Random housing	Caregiver/ investigator blinding	Random outcome assessment	Outcome-assessor blinding	Incomplete outcome data	Selective reporting	Other bias
**Rated cells, n**	67	67	67	67	67	67	67	67	67	67
**Low risk, n (%)**	0 (0.0)	13 (19.4)	0 (0.0)	0 (0.0)	0 (0.0)	0 (0.0)	0 (0.0)	21 (31.3)	6 (9.0)	0 (0.0)
**Unclear risk, n (%)**	65 (97.0)	53 (79.1)	67 (100.0)	67 (100.0)	55 (82.1)	67 (100.0)	59 (88.1)	27 (40.3)	50 (74.6)	6 (9.0)
**High risk, n (%)**	2 (3.0)	1 (1.5)	0 (0.0)	0 (0.0)	12 (17.9)	0 (0.0)	8 (11.9)	19 (28.4)	11 (16.4)	61 (91.0)

Note: Percentages were calculated among rated cells (L, U, or H) within each domain; N/A and cells without a source rating were excluded from the denominator. The concise version includes the 67 studies with at least one SYRCLE rating; 89 source rows without a SYRCLE rating were omitted.
