# Supplementary material for: The Role of Circulating MicroRNAs as Biomarkers and Therapeutic Targets in Venous Thromboembolism: A Systematic Review
Source: J Clin Med. 2026 Aug 14;15(16):6306. doi: 10.3390/jcm15166306 (PMC13513252; doi:10.3390/jcm15166306)
Supplement: Supplementary file 1 [file jcm-15-06306-s001.zip › Supplementary Table S4.pdf]

**Supplementary Table S4a.** Quality assessment of case-control studies using the Newcastle-Ottawa Scale (NOS).

| Study | Selection | | | | Comparability | | Exposure | | | Total score |
|---|---|---|---|---|---|---|---|---|---|---|
| | S1 | S2 | S3 | S4 | C1 | C2 | E1 | E2 | E3 | |
| Zhou, 2024 [33] | ★ | - | - | ★ | - | - | ★ | ★ | - | **4/9** |
| Wang, 2025 [34] | ★ | - | - | - | - | - | ★ | ★ | - | **3/9** |
| Xu, 2022 [36] | ★ | - | ★ | - | ★ | - | ★ | ★ | - | **5/9** |
| Huang, 2023 [37] | ★ | - | ★ | ★ | ★ | - | ★ | ★ | - | **6/9** |
| Han, 2023 [39] | ★ | - | ★ | ★ | - | - | ★ | ★ | - | **5/9** |
| Wang, 2025 [41] | ★ | - | ★ | - | ★ | ★ | ★ | ★ | - | **6/9** |
| Qin, 2015 [42] | ★ | ★ | ★ | ★ | ★ | - | ★ | ★ | - | **7/9** |
| Jin, 2019 [45] | ★ | - | - | - | - | - | ★ | ★ | - | **3/9** |
| Tian, 2022 [46] | - | - | ★ | - | - | - | ★ | ★ | - | **3/9** |
| Jiang, 2017 [49] | ★ | ★ | ★ | - | ★ | - | ★ | ★ | - | **6/9** |
| Srivastava, 2019 [50] | ★ | - | ★ | ★ | ★ | - | ★ | ★ | - | **6/9** |
| Zhang, 2020 [53] | ★ | ★ | - | - | ★ | - | ★ | ★ | - | **5/9** |
| Wang, 2016 [55] | ★ | ★ | ★ | ★ | ★ | - | ★ | ★ | - | **7/9** |
| Xiao, 2024 [56] | ★ | - | ★ | ★ | ★ | ★ | ★ | ★ | - | **7/9** |
| Zhang, 2024 [61] | ★ | - | - | - | ★ | - | ★ | ★ | - | **4/9** |
| Kong, 2016 [62] | ★ | - | - | - | ★ | - | ★ | ★ | - | **4/9** |
| Qiao, 2024 [67] | - | - | - | - | - | - | ★ | ★ | - | **2/9** |
| Lou, 2022 [70] | ★ | - | - | - | - | - | ★ | ★ | - | **3/9** |
| Li, 2017 [71] | ★ | - | - | - | - | - | ★ | ★ | - | **3/9** |
| Rahmanpour, 2022 [72] | ★ | - | - | - | ★ | ★ | ★ | ★ | - | **5/9** |
| Lou, 2022 [73] | - | - | - | - | - | - | ★ | ★ | - | **2/9** |
| Sun, 2020 [74] | ★ | - | ★ | - | - | - | ★ | ★ | - | **4/9** |
| Cao, 2022 [75] | ★ | - | - | - | - | - | ★ | ★ | - | **3/9** |
| Zhang, 2020 [76] | - | - | - | - | - | - | ★ | ★ | - | **2/9** |
| Li, 2020 [77] | - | - | - | - | - | - | ★ | ★ | - | **2/9** |
| Ou, 2020 [82] | - | - | ★ | - | - | - | ★ | ★ | - | **3/9** |
| Jha, 2021 [86] | ★ | - | ★ | - | ★ | ★ | ★ | ★ | - | **6/9** |
| Sahu, 2017 [87] | ★ | - | - | - | - | - | ★ | ★ | - | **3/9** |
| Antoun, 2024 [88] | ★ | ★ | ★ | ★ | ★ | - | ★ | ★ | ★ | **8/9** |
| Wang, 2024 [89] | ★ | - | - | - | ★ | - | ★ | ★ | - | **4/9** |

| Study | Selection | | | | Comparability | | Exposure | | | Total score |
|---|---|---|---|---|---|---|---|---|---|---|
| | S1 | S2 | S3 | S4 | C1 | C2 | E1 | E2 | E3 | |
| Wang, 2019 [91] | ★ | - | - | - | ★ | - | ★ | ★ | - | **4/9** |
| Zhang, 2024 [95] | ★ | - | - | - | ★ | - | - | ★ | - | **3/9** |
| Yang, 2023 [96] | ★ | - | - | - | ★ | - | ★ | ★ | - | **4/9** |
| Ding et al, 2022 [99] | ★ | - | ★ | ★ | - | - | ★ | ★ | - | **5/9** |
| Chen et al, 2023 [100] | - | - | - | - | - | - | ★ | ★ | - | **2/9** |
| Wang et al, 2023 [102] | ★ | - | ★ | ★ | ★ | ★ | ★ | ★ | - | **7/9** |
| Sun et al, 2020 [104] | ★ | - | ★ | - | ★ | - | ★ | ★ | - | **5/9** |
| Xu et al, 2022 [105] | ★ | - | ★ | - | ★ | ★ | ★ | ★ | - | **6/9** |
| Pan et al, 2021 [107] | ★ | - | - | - | - | - | ★ | ★ | - | **3/9** |
| Zhang et al, 2025 [108] | ★ | - | ★ | ★ | ★ | ★ | ★ | ★ | - | **7/9** |
| Zhang et al, 2020 [109] | ★ | - | - | - | ★ | ★ | ★ | ★ | - | **5/9** |
| Marchand et al, 2012 [113] | ★ | - | ★ | ★ | - | - | ★ | ★ | - | **5/9** |
| Chang et al, 2019 [114] | ★ | - | ★ | ★ | - | - | ★ | ★ | - | **5/9** |
| Kong et al, 2016 [115] | ★ | - | - | - | ★ | - | ★ | ★ | - | **4/9** |
| Chu et al, 2022 [119] | ★ | - | - | - | ★ | ★ | ★ | ★ | - | **5/9** |
| Wang et al, 2018 [121] | ★ | - | ★ | ★ | ★ | - | ★ | ★ | - | **6/9** |
| Lu et al, 2021 [124] | ★ | - | ★ | - | - | - | ★ | ★ | - | **4/9** |
| Wang et al., 2023 [125] | - | - | ★ | - | ★ | ★ | ★ | ★ | - | **5/9** |
| Zhang et al., 2025 [128] | - | - | ★ | - | - | - | ★ | ★ | - | **3/9** |
| Pan et al., 2025 [129] | - | - | ★ | - | ★ | ★ | ★ | ★ | - | **5/9** |
| Shi et al, 2025 [130] | ★ | - | ★ | ★ | - | - | ★ | ★ | - | **5/9** |
| Fabro, 2021 [133] | ★ | - | - | - | ★ | - | ★ | ★ | - | **4/9** |
| Wang, 2018 [136] | ★ | - | - | ★ | ★ | ★ | ★ | ★ | - | **6/9** |
| Zhou, 2016 [137] | ★ | - | - | ★ | ★ | ★ | ★ | ★ | - | **6/9** |
| Mao, 2020 [138] | - | - | - | ★ | - | - | ★ | ★ | - | **3/9** |
| Xiao, 2011 [139] | ★ | ★ | ★ | ★ | - | - | ★ | ★ | - | **6/9** |
| Zhou, 2021 [140] | ★ | - | - | ★ | - | - | ★ | ★ | - | **4/9** |
| Liu, 2018 [141] | ★ | - | - | ★ | - | - | ★ | ★ | - | **4/9** |
| Tang, 2024 [142] | ★ | - | - | ★ | - | - | ★ | ★ | - | **4/9** |
| Sun, 2019 [143] | ★ | - | - | ★ | ★ | ★ | ★ | ★ | - | **6/9** |
| Kessler, 2016 [144] | ★ | - | ★ | ★ | ★ | ★ | ★ | ★ | - | **7/9** |

| Study | Selection | | | | Comparability | | Exposure | | | Total score |
|---|---|---|---|---|---|---|---|---|---|---|
| | S1 | S2 | S3 | S4 | C1 | C2 | E1 | E2 | E3 | |
| Peng, 2020 [145] | ★ | - | ★ | ★ | - | - | ★ | ★ | - | **5/9** |
| Li, 2022 [147] | ★ | - | - | ★ | - | - | ★ | ★ | - | **4/9** |
| Nafady, 2022 [151] | ★ | - | ★ | - | - | - | ★ | ★ | - | **4/9** |
| Wu, 2024 [153] | ★ | - | ★ | - | ★ | ★ | ★ | ★ | - | **6/9** |
| Wang, 2024 [158] | - | - | - | ★ | ★ | ★ | ★ | ★ | - | **5/9** |
| Jian, 2021 [160] | ★ | - | - | ★ | ★ | - | ★ | ★ | - | **5/9** |
| Erhart, 2024 [161] | ★ | ★ | ★ | ★ | ★ | ★ | ★ | ★ | - | **8/9** |
| Zhang, 2023 [162] | ★ | - | ★ | - | ★ | ★ | ★ | ★ | - | **6/9** |
| Li, 2023 [163] | ★ | - | - | ★ | ★ | ★ | ★ | ★ | - | **6/9** |
| Gabler, 2021 [164] | ★ | - | - | ★ | ★ | ★ | ★ | ★ | - | **6/9** |
| Zeng et al, 2019 [165] | - | - | - | ★ | ★ | - | ★ | ★ | - | **4/9** |
| Wang et al, 2020 [168] | - | ★ | ★ | - | - | - | ★ | ★ | - | **4/9** |
| Starikova et al., 2015 [173] | ★ | - | ★ | ★ | ★ | - | ★ | ★ | - | **6/9** |
| Wang et al., 2019 [174] | ★ | ★ | ★ | ★ | ★ | - | ★ | ★ | ★ | **8/9** |
| Kim et al., 2019 [175] | ★ | ★ | - | ★ | ★ | ★ | ★ | ★ | - | **7/9** |
| Ko et al., 2019 [176] | ★ | - | - | ★ | ★ | ★ | ★ | ★ | - | **6/9** |
| Rodriguez-Rius et al., 2020 [178] | - | - | ★ | ★ | ★ | ★ | ★ | ★ | - | **6/9** |
| Oto et al., 2020 [179] | ★ | ★ | ★ | ★ | ★ | - | ★ | ★ | - | **7/9** |
| Anjis et al, 2022 [180] | - | - | ★ | ★ | ★ | ★ | ★ | ★ | - | **6/9** |
| Fang et al., 2022 [181] | ★ | - | - | - | - | - | ★ | ★ | - | **3/9** |
| Su et al., 2021 [183] | ★ | - | - | - | ★ | - | ★ | ★ | - | **4/9** |
| Lin et al, 2025 [187] | - | - | - | - | - | - | ★ | ★ | - | **2/9** |

**Note:** S1, adequacy of case definition; S2, representativeness of cases; S3, selection of controls; S4, definition of controls; C1, control for the major confounder; C2, control for additional confounders; E1, ascertainment of exposure; E2, same ascertainment method for cases and controls; E3, non-response rate. ★ indicates that an item was awarded; - indicates that no item was awarded. Maximum score: 9. Studies for which the case-control NOS was not applicable were omitted.

**Supplementary Table S4b.** Quality assessment of cohort studies using the Newcastle-Ottawa Scale (NOS).

| Study | Selection | | | | Comparability | | Outcome | | | Total score |
|---|---|---|---|---|---|---|---|---|---|---|
| | S1 | S2 | S3 | S4 | C1 | C2 | O1 | O2 | O3 | |
| Zhou, 2024 [33] | - | ★ | ★ | ★ | ★ | - | - | ★ | ★ | **6/9** |
| Xie, 2016 [35] | - | ★ | ★ | ★ | - | - | ★ | - | ★ | **5/9** |
| Zhou, 2024 [47] | - | ★ | ★ | ★ | ★ | ★ | ★ | - | ★ | **7/9** |
| Tang, 2021 [52] | - | ★ | ★ | ★ | - | - | ★ | - | ★ | **5/9** |
| Wang, 2022 [57] | - | ★ | ★ | - | ★ | - | ★ | ★ | ★ | **6/9** |
| Guo, 2025 [59] | - | ★ | ★ | - | ★ | - | ★ | - | ★ | **5/9** |
| Du, 2019 [66] | - | ★ | ★ | ★ | ★ | ★ | - | ★ | - | **6/9** |
| Liang, 2020 [69] | - | ★ | ★ | ★ | - | - | ★ | ★ | - | **5/9** |
| Zhang, 2021 [94] | - | ★ | ★ | - | - | - | - | - | - | **2/9** |
| Oto et al., 2020 [154] | ★ | ★ | ★ | ★ | ★ | - | ★ | ★ | ★ | **8/9** |
| Rossetti, 2021 [156] | - | ★ | ★ | ★ | - | - | - | ★ | - | **4/9** |
| Morelli et al, 2024 [170] | ★ | ★ | ★ | ★ | ★ | ★ | ★ | ★ | ★ | **9/9** |
| Teixeira-Costa et al, 2025 [171] | - | ★ | ★ | - | - | - | ★ | ★ | - | **4/9** |
| Ciccacci et al., 2015 [172] | ★ | ★ | ★ | - | ★ | ★ | ★ | ★ | ★ | **8/9** |
| Thibord et al., 2020 [177] | - | ★ | ★ | ★ | ★ | - | - | ★ | - | **5/9** |
| Ten Cate et al., 2023 [182] | - | ★ | ★ | ★ | ★ | ★ | ★ | ★ | - | **7/9** |

**Note:** S1, representativeness of the exposed cohort; S2, selection of the non-exposed cohort; S3, ascertainment of exposure; S4, outcome absent at baseline; C1, control for the major confounder; C2, control for additional confounders; O1, assessment of outcome; O2, follow-up long enough for the outcome; O3, adequacy of follow-up. ★ indicates that an item was awarded; - indicates that no item was awarded. Maximum score: 9. Studies for which the cohort NOS was not applicable were omitted.
