# Supplementary material for: The Role of Circulating MicroRNAs as Biomarkers and Therapeutic Targets in Venous Thromboembolism: A Systematic Review
Source: J Clin Med. 2026 Aug 14;15(16):6306. doi: 10.3390/jcm15166306 (PMC13513252; doi:10.3390/jcm15166306)
Supplement: Supplementary file 1 [file jcm-15-06306-s001.zip › Supplementary Table S5.pdf]

Supplementary Table S5. Experimental and clinical study-design components represented in the included publications.

Study (first author, year)	In vitro	Animal model	Human clinical study (case-control/cohort)
Feng, 2022 [32]	Yes	Yes	NA
Zhou, 2024 [33]	Yes	NA	Yes
Wang, 2025 [34]	Yes	Yes	Yes
Xie, 2016 [35]	Yes	NA	Yes
Xu, 2022 [36]	Yes	NA	Yes
Huang, 2023 [37]	Yes	NA	Yes
Yu, 2023 [38]	Yes	Yes	NA
Han, 2023 [39]	Yes	NA	Yes
Zhang, 2018 [40]	Yes	Yes	NA
Wang, 2025 [41]	Yes	NA	Yes
Qin, 2015 [42]	Yes	NA	Yes
Mo, 2016 [43]	Yes	NA	NA
Qin, 2018 [44]	Yes	NA	NA
Jin, 2019 [45]	Yes	NA	Yes
Tian, 2022 [46]	Yes	NA	Yes
Zhou, 2024 [47]	Yes	NA	Yes
Li, 2022 [48]	Yes	Yes	NA
Jiang, 2017 [49]	Yes	NA	Yes
Srivastava, 2019 [50]	Yes	NA	Yes
Srivastava, 2024 [51]	Yes	Yes	NA
Tang, 2021 [52]	Yes	NA	Yes
Zhang, 2020 [53]	Yes	Yes	Yes
Wang, 2022 [54]	Yes	NA	NA
Wang, 2016 [55]	Yes	NA	Yes
Xiao, 2024 [56]	Yes	NA	Yes

Study (first author, year)	In vitro	Animal model	Human clinical study (case-control/cohort)
Wang, 2022 [57]	Yes	NA	Yes
Wang, 2021 [58]	Yes	Yes	NA
Guo, 2025 [59]	Yes	NA	Yes
Lu, 2019 [60]	Yes	Yes	NA
Zhang, 2024 [61]	Yes	Yes	Yes
Kong, 2016 [62]	Yes	Yes	Yes
Zhu, 2020 [63]	Yes	NA	NA
Zhou, 2020 [64]	Yes	Yes	NA
Wang, 2018 [65]	Yes	NA	NA
Du, 2019 [66]	Yes	Yes	Yes
Qiao, 2024 [67]	Yes	Yes	Yes
Wang, 2026 [68]	Yes	Yes	NA
Liang, 2020 [69]	Yes	NA	Yes
Lou, 2022 [70]	Yes	NA	Yes
Li, 2017 [71]	Yes	NA	Yes
Rahmanpour, 2022 [72]	Yes	NA	Yes
Lou, 2022 [73]	Yes	Yes	Yes
Sun, 2020 [74]	Yes	Yes	Yes
Cao, 2022 [75]	Yes	NA	Yes
Zhang, 2020 [76]	Yes	NA	Yes
Li, 2020 [77]	Yes	NA	Yes
Yang, 2022 [78]	Yes	Yes	NA
Meng, 2015 [79]	Yes	Yes	NA
Chen, 2016 [80]	Yes	Yes	NA
Zhu, 2023 [81]	Yes	NA	NA
Ou, 2020 [82]	Yes	Yes	Yes

Study (first author, year)	In vitro	Animal model	Human clinical study (case-control/cohort)
Feng, 2022 [83]	Yes	Yes	NA
Chen, 2025 [84]	Yes	Yes	NA
Zhang, 2020 [85]	Yes	NA	NA
Jha, 2021 [86]	Yes	NA	Yes
Sahu, 2017 [87]	Yes	Yes	Yes
Antoun, 2024 [88]	Yes	NA	Yes
Wang, 2024 [89]	Yes	Yes	Yes
Wang, 2014 [90]	Yes	Yes	NA
Wang, 2019 [91]	Yes	Yes	Yes
Du, 2020 [92]	Yes	Yes	NA
He, 2023 [93]	Yes	Yes	NA
Zhang, 2021 [94]	NA	NA	Yes
Zhang, 2024 [95]	Yes	NA	Yes
Yang, 2023 [96]	Yes	NA	Yes
Lu, 2022 [97]	Yes	Yes	NA
Ni, 2023 [98]	Yes	Yes	NA
Ding, 2022 [99]	Yes	Yes	Yes
Chen, 2023 [100]	Yes	NA	Yes
Sun, 2019 [101]	Yes	Yes	NA
Wang, 2023 [102]	Yes	NA	Yes
Li, 2020 [103]	NA	Yes	NA
Sun, 2020 [104]	Yes	Yes	Yes
Xu, 2022 [105]	NA	NA	Yes
Luo, 2025 [106]	Yes	Yes	NA
Pan, 2021 [107]	Yes	Yes	Yes
Zhang, 2025 [108]	Yes	NA	Yes

Study (first author, year)	In vitro	Animal model	Human clinical study (case-control/cohort)
Zhang, 2020 [109]	Yes	Yes	Yes
Pan, 2022 [110]	Yes	Yes	NA
Yang, 2020 [111]	Yes	Yes	NA
Ai, 2018 [112]	Yes	Yes	NA
Marchand, 2012 [113]	Yes	NA	Yes
Chang, 2019 [114]	NA	NA	Yes
Kong, 2016 [115]	Yes	Yes	Yes
Fan, 2024 [116]	Yes	Yes	NA
Tang, 2018 [117]	Yes	Yes	NA
Xu, 2025 [118]	Yes	Yes	NA
Chu, 2022 [119]	Yes	Yes	Yes
Zhu, 2023 [120]	Yes	NA	NA
Wang, 2018 [121]	NA	NA	Yes
Qui, 2024 [122]	Yes	Yes	NA
Li, 2018 [123]	Yes	Yes	NA
Lu, 2021 [124]	Yes	Yes	Yes
Wang, 2023 [125]	NA	NA	Yes
Jin, 2017 [126]	NA	Yes	NA
Hao, 2024 [127]	NA	Yes	NA
Zhang, 2025 [128]	NA	NA	Yes
Pan, 2025 [129]	NA	NA	Yes
Shi, 2025 [130]	NA	NA	Yes
Liu, 2020 [131]	Yes	Yes	NA
Zhang, 2022 [132]	Yes	NA	NA
Fabro, 2021 [133]	NA	NA	Yes
Liang, 2021 [134]	NA	Yes	NA

Study (first author, year)	In vitro	Animal model	Human clinical study (case-control/cohort)
Shen, 2025 [135]	Yes	Yes	NA
Wang, 2018 [136]	NA	NA	Yes
Zhou, 2016 [137]	NA	Yes	Yes
Mao, 2020 [138]	Yes	Yes	Yes
Xiao, 2011 [139]	NA	NA	Yes
Zhou, 2021 [140]	NA	NA	Yes
Liu, 2018 [141]	NA	NA	Yes
Tang, 2024 [142]	Yes	NA	Yes
Sun, 2019 [143]	NA	Yes	Yes
Kessler, 2016 [144]	NA	NA	Yes
Peng, 2020 [145]	NA	NA	Yes
Yang, 2019 [146]	Yes	Yes	NA
Li, 2022 [147]	Yes	Yes	Yes
Song, 2024 [148]	Yes	Yes	NA
Chen, 2020 [149]	Yes	Yes	NA
Yang, 2025 [150]	NA	Yes	NA
Nafady, 2022 [151]	NA	NA	Yes
Jiang, 2024 [152]	NA	Yes	NA
Wu, 2024 [153]	Yes	NA	Yes
Oto, 2020 [154]	NA	NA	Yes
Zhu, 2022 [155]	Yes	Yes	NA
Rossetti, 2021 [156]	NA	NA	Yes
Cao, 2018 [157]	NA	NA	NA
Wang, 2024 [158]	Yes	NA	Yes
Fazzalari, 2021 [159]	Yes	Yes	NA
Jian, 2021 [160]	Yes	Yes	Yes

Study (first author, year)	In vitro	Animal model	Human clinical study (case-control/cohort)
Erhart, 2024 [161]	NA	NA	Yes
Zhang, 2023 [162]	NA	NA	Yes
Li, 2023 [163]	Yes	NA	Yes
Gabler, 2021 [164]	NA	NA	Yes
Zeng, 2019 [165]	Yes	Yes	Yes
Tian, 2021 [166]	Yes	NA	NA
Lan, 2022 [167]	Yes	NA	NA
Wang, 2020 [168]	NA	NA	Yes
Chen, 2021 [169]	NA	NA	NA
Morelli, 2024 [170]	NA	NA	Yes
Teixeira-Costa, 2025 [171]	NA	NA	Yes
Ciccacci, 2015 [172]	NA	NA	Yes
Starikova, 2015 [173]	NA	NA	Yes
Wang, 2019 [174]	NA	NA	Yes
Kim, 2019 [175]	NA	NA	Yes
Ko, 2019 [176]	NA	NA	Yes
Thibord, 2020 [177]	NA	NA	Yes
Rodriguez-Rius, 2020 [178]	NA	NA	Yes
Oto, 2020 [179]	NA	NA	Yes
Anjis, 2022 [180]	NA	NA	Yes
Fang, 2022 [181]	NA	NA	Yes
Ten Cate, 2023 [182]	NA	NA	Yes
Su, 2021 [183]	NA	NA	Yes
Rouka, 2023 [184]	NA	NA	NA
Chancal, 2024 [185]	NA	NA	NA
Zhang, 2024 [186]	NA	NA	NA

Study (first author, year)	In vitro	Animal model	Human clinical study (case-control/cohort)
Lin, 2025 [187]	NA	NA	Yes
**Total, n (%)**	**106 (67.9)**	**67 (42.9)**	**98 (62.8)**

Note: "Yes" indicates that the publication included the corresponding study-design component. NA, not applicable. Categories are not mutually exclusive.
